# MOTS-c modulates skeletal muscle function by directly binding and activating CK2

**DOI:** 10.1016/j.isci.2024.111212

**Published:** 2024-10-19

**Authors:** Hiroshi Kumagai, Su-Jeong Kim, Brendan Miller, Hirofumi Zempo, Kumpei Tanisawa, Toshiharu Natsume, Shin Hyung Lee, Junxiang Wan, Naphada Leelaprachakul, Michi Emma Kumagai, Ricardo Ramirez, Hemal H. Mehta, Kevin Cao, Tae Jung Oh, James A. Wohlschlegel, Jihui Sha, Yuichiro Nishida, Noriyuki Fuku, Shohei Dobashi, Eri Miyamoto-Mikami, Mizuki Takaragawa, Mizuho Fuku, Toshinori Yoshihara, Hisashi Naito, Ryoko Kawakami, Suguru Torii, Taishi Midorikawa, Koichiro Oka, Megumi Hara, Chiharu Iwasaka, Yosuke Yamada, Yasuki Higaki, Keitaro Tanaka, Kelvin Yen, Pinchas Cohen

**Affiliations:** 1 The Leonard Davis School of Gerontology, University of Southern California, Los Angeles, CA, USA; 2 Department of Administrative Nutrition, Faculty of Health and Nutrition, Tokyo Seiei College, Tokyo, Japan; 3 Faculty of Sport Sciences, Waseda University, Saitama, Japan; 4 Faculty of Medicine, Tokai University, Kanagawa, Japan; 5 Department of Human Genetics, David Geffen School of Medicine, University of California, Los Angeles, CA, USA; 6 Department of Internal Medicine, Seoul National University College of Medicine and Seoul National University Bundang Hospital, Seongnam, South Korea; 7 Department of Biological Chemistry, David Geffen School of Medicine, University of California, Los Angeles, CA, USA; 8 Department of Preventive Medicine, Faculty of Medicine, Saga University, Saga, Japan; 9 Graduate School of Health and Sports Science, Juntendo University, Chiba, Japan; 10 Institute of Health and Sport Sciences, University of Tsukuba, Ibaraki, Japan; 11 Tsudanuma Central General Hospital, Chiba, Japan; 12 Physical Fitness Research Institute, Meiji Yasuda Life Foundation of Health and Welfare, Tokyo, Japan; 13 College of Health and Welfare, J.F. Oberlin University, Tokyo, Japan; 14 Department of Physical Activity Research, National Institutes of Biomedical Innovation, Health and Nutrition, Osaka, Japan; 15 Sports and Health Sciences, Graduate School of Biomedical Engineering, Tohoku University, Miyagi, Japan; 16 Medicine and Science in Sports and Exercise, Graduate School of Medicine, Tohoku University, Miyagi, Japan; 17 Laboratory of Exercise Physiology, Faculty of Sports and Health Science, Fukuoka University, Fukuoka, Japan

**Keywords:** Physiology, cell biology

## Abstract

MOTS-c is a mitochondrial microprotein that improves metabolism. Here, we demonstrate CK2 is a direct and functional target of MOTS-c. MOTS-c directly binds to CK2 and activates it in cell-free systems. MOTS-c administration to mice prevented skeletal muscle atrophy and enhanced muscle glucose uptake, which were blunted by suppressing CK2 activity. Interestingly, the effects of MOTS-c are tissue-specific. Systemically administered MOTS-c binds to CK2 in fat and muscle, yet stimulates CK2 activity in muscle while suppressing it in fat by differentially modifying CK2-interacting proteins. Notably, a naturally occurring MOTS-c variant, K14Q MOTS-c, has reduced binding to CK2 and does not activate it or elicit its effects. Male K14Q MOTS-c carriers exhibited a higher risk of sarcopenia and type 2 diabetes (T2D) in an age- and physical-activity-dependent manner, whereas females had an age-specific reduced risk of T2D. Altogether, these findings provide evidence that CK2 is required for MOTS-c effects.

## Highlights

• MOTS-c directly binds to CK2 and regulates its activity in a tissue-specific manner
• MOTS-c modulates glucose metabolism and muscle mass by activating CK2
• A naturally occurring MOTS-c variant is a risk of type 2 diabetes and sarcopenia in men
• Physical activity normalizes the negative effects of the MOTS-c variant in men

## Graphical abstract

**Figure:**
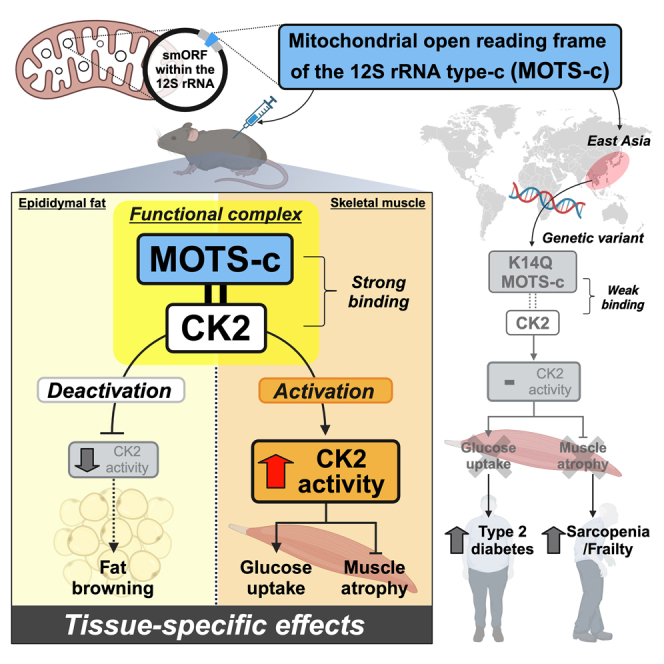

## Introduction

MOTS-c (mitochondrial open reading frame of the 12S ribosomal RNA type-c), a 16-amino acid microprotein encoded in a small open reading frame (smORF) within the 12S rRNA region of the mitochondrial DNA (mtDNA), was identified in 2015 and has been recognized as a metabolic regulator.^1^^,^^2^^,^^3^^,^^4^^,^^5^ MOTS-c is expressed in multiple tissues, including skeletal muscle,^1^^,^^6^^,^^7^ and is induced by exercise^6^^,^^8^ and has exercise mimetic effects.^1^^,^^6^^,^^9^^,^^10^ For instance, MOTS-c administration improves glucose metabolism,^1^^,^^11^ increases exercise endurance,^6^^,^^9^ and prevents muscle atrophy.^6^^,^^10^^,^^12^ Additionally, a naturally occurring MOTS-c variant (rs111033358),^13^ m.1382A>C causing K14Q amino acid substitution, increases type 2 diabetes (T2D) risk and modulates skeletal muscle fiber type composition and physical performance.^11^^,^^14^ However, the direct molecular targets of MOTS-c in skeletal muscle have not been fully elucidated.

One of the potential molecular targets of MOTS-c is protein kinase CK2 (formerly called casein kinase 2). We have previously observed an increased CK2 activity in the MOTS-c-treated mouse skeletal muscle as well as its downstream target AKT.^10^ CK2, a tetrameric protein kinase with two catalytic alpha and two regulatory beta subunits, is a ubiquitous and highly conserved serine-threonine kinase. CK2 targets over 300 protein substrates^15^ and involves in multiple functions, including energy metabolism and cell growth.^16^ More specifically, CK2 modulates glucose uptake by regulating the AKT signaling pathway in multiple tissues and cells.^17^^,^^18^ Additionally, in the skeletal muscle, CK2 involves in muscle homeostasis, such as myogenesis, regeneration, and fiber type composition.^19^^,^^20^^,^^21^^,^^22^^,^^23^ Since those established CK2-related phenotypes overlapped with MOTS-c functions and increased CK2 activity was observed in MOTS-c-treated mouse skeletal muscle, we hypothesized that CK2 was a direct binding partner and functional target of the mitochondrial microprotein MOTS-c. Furthermore, we also hypothesized that the naturally occurring MOTS-c variant, m.1382A>C causing a K14Q amino acid replacement, could modify skeletal muscle function by differentially interacting with CK2.

Here, we examined a direct interaction of CK2 and MOTS-c, including both wild-type (WT) and K14Q MOTS-c, *in vitro* and *in vivo*. We also examined the influence of the naturally occurring MOTS-c variant, m.1382A>C causing K14Q amino acid replacement, on skeletal muscle mass, function (e.g., strength), and the prevalence of T2D in human subjects.

## Results

### MOTS-c increases CK2 activity in the skeletal muscle

**Figure 1:**
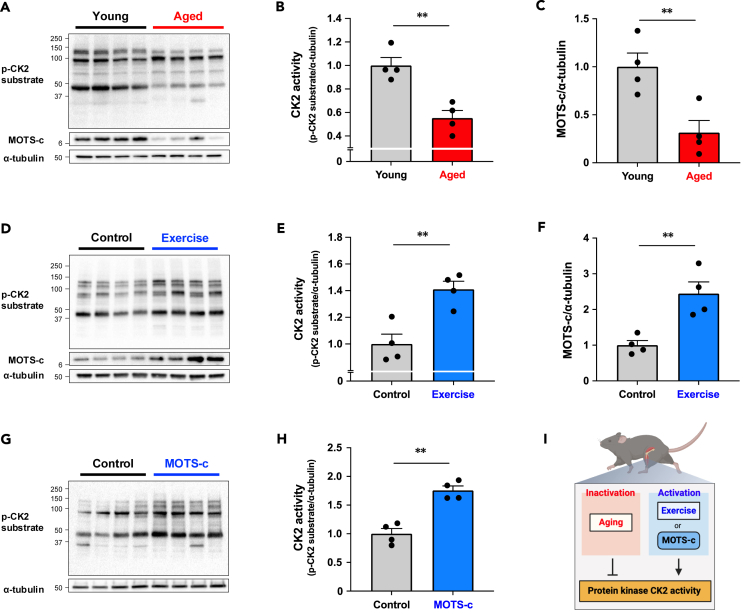
Endogenous and exogenous MOTS-c regulates CK2 activity in the skeletal muscle (A–C) CK2 activity assessed by detecting endogenous proteins containing a pS/pTDXE motif and MOTS-c expression levels in gastrocnemius muscle from young control (2 months) and aged (22 months) mice (*n* = 4 per group). (D–F) Effect of 4 weeks of voluntary wheel running exercise on CK2 activity and MOTS-c expression levels in gastrocnemius muscle from young mice (*n* = 4 per group). (G and H) Effect of 8 weeks of MOTS-c administration (5 mg/kg/day) on CK2 activity in quadriceps muscle from high-fat-diet (HFD)-fed mice (*n* = 4 per group). (I) Summary of MOTS-c and CK2 activity in the skeletal muscle. Data are represented as mean ± SEM for (B, C, E, F, and H). ∗∗*p* < 0.01.

We previously demonstrated that exogenous MOTS-c increased CK2 activity as assessed by the detection of endogenous proteins containing a pS/pTDXE motif, which is a CK2 phosphorylation consensus sequence, in the skeletal muscle.^10^ First, we examined the correlations between expression levels of MOTS-c, CK2α and CK2β, and CK2 activity in the skeletal muscle. Aging was associated with lower MOTS-c, CK2α, and CK2β expression levels, as well as lower CK2 activity in skeletal muscle of mice (Figures 1A–1C; Figure S1). We also assessed phospho-AKT Ser129 and phospho-CDC37, direct CK2 substrates, as alternative markers of CK2 activity.^24^ Aging decreased both total AKT and total CDC37 levels. Phospho-CDC37/total CDC37 was significantly decreased in aged muscle, whereas there was no difference in phospho-AKT/total AKT (Figure S1). On the other hand, MOTS-c expression and CK2 activity were increased by 4 weeks of wheel running exercise (Figures 1D–1F) without changing CK2α and CK2β expression levels (Figure S2). Phospho-CDC37/total CDC37 was increased in the exercised group, whereas phospho-AKT/total AKT did not change since total AKT levels were higher in the exercised group (Figure S2). Although the effect of exercise training on CK2 activity is not well understood, a study has suggested that a single bout of cycling exercise increases CK2α activity in individuals with obesity.^25^ Thus, our present observations corroborate this finding and provide novel insights into the impact of long-term exercise training on muscle CK2 activity. We also validated our previous observation^10^ and confirmed that 8 weeks of MOTS-c treatment increased CK2 activity in skeletal muscle from high-fat-diet-fed mice (Figures 1G and 1H). Although expression levels of CK2β increased by MOTS-c treatment, CK2α levels did not change (Figure S3). MOTS-c treatment increased both phospho-AKT/total AKT and phospho-CDC37/total CDC37 levels in skeletal muscle (Figure S3). These findings suggest that MOTS-c expression levels could correlate with CK2 activity in the skeletal muscle, and MOTS-c could be directly regulating CK2 activity (Figure 1I).

### MOTS-c directly binds to the CK2α subunit and increases CK2 activity in cell-free systems

**Figure 2:**
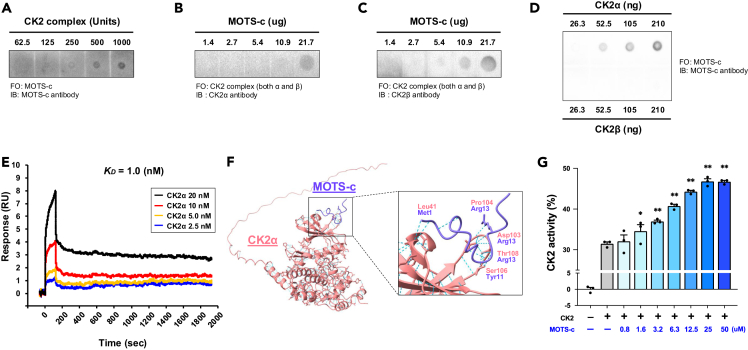
MOTS-c directly binds and activates CK2 in cell-free systems (A–C) Dot blot assays with (A) CK2 complex (contains both CK2α and CK2β subunits) immobilized, MOTS-c flowed over the membrane, and detected by MOTS-c antibody. (B–C) MOTS-c immobilized, CK2 complex flowed over the membrane, and detected by CK2α (B) and CK2β (C) antibodies. (D) CK2α or CK2β immobilized, MOTS-c flowed over the membrane, and detected by MOTS-c antibody. FO, flow over; IB, immunoblotting. (E): Surface plasmon resonance (Biacore assay) of MOTS-c (10 μg/mL) and CK2α (2.5 nM, 5.0 nM, 10 nM, and 20 nM). MOTS-c was immobilized on the sensor chip and CK2α flowed over the sensor chip. *K*_*D*_, dissociation constant. (F) Molecular docking simulation of the binding between MOTS-c and CK2α by using AlphaFold2. (G) CK2 activity assessed by kinase activity assay with/without MOTS-c in cell-free condition. A different dose of MOTS-c (0–100 μM) was used for the assay. Data are represented as mean ± SEM for (G). ∗*p* < 0.05, ∗∗*p* < 0.01 versus CK2 without MOTS-c group.

We next examined the direct interaction between MOTS-c and CK2. First, we checked the binding between MOTS-c and CK2 by using a dot blot assay. We observed that MOTS-c directly binds to CK2, containing both CK2α and CK2β subunits (Figures 2A–2C; Figures S4A–S4C). Next, we used each CK2α and CK2β subunit to identify which subunit MOTS-c bound to. Although we detected a weak signal in the negative controls, we observed that MOTS-c bound to the CK2α subunit, but not the CK2β subunit (Figure 2D; Figure S4D). We confidently confirmed this binding between MOTS-c and CK2α by using a surface plasmon resonance assay (also called a Biacore assay), and the dissociation constant (*K*_*D*_) was 1 nM, which indicates MOTS-c strongly binds to CK2α (Figure 2E; Figures S5A and S5B). Since the surface plasmon resonance assay is non-antibody-based binding assay, the observed binding between MOTS-c and CK2α is a reliable binding, not a non-specific binding. To understand more about the binding, we simulated the MOTS-c/CK2α complex by using AlphaFold2-Multimer.^26^^,^^27^^,^^28^ The simulation suggested that MOTS-c and CK2α interact at Met1 (MOTS-c)-Leu41 (CK2α), Arg13 (MOTS-c)-Asp103 (CK2α), Arg13 (MOTS-c)-Pro104 (CK2α), Tyr11 (MOTS-c)-Ser106 (CK2α), and Arg13 (MOTS-c)-Thr108 (CK2α) (Figure 2F). Then, we examined if MOTS-c increased the ability of CK2 to phosphorylate its substrates in a cell-free condition by using a kinase activity assay with a commercially available specific CK2 substrate. We found that MOTS-c increased the ability of CK2 to phosphorylate its substrate in a dose-dependent manner (Figure 2G). Those results demonstrate that MOTS-c directly binds to CK2α and increases CK2 activity.

### MOTS-c differentially modulates CK2 activity in skeletal muscle and adipose tissue

**Figure 3:**
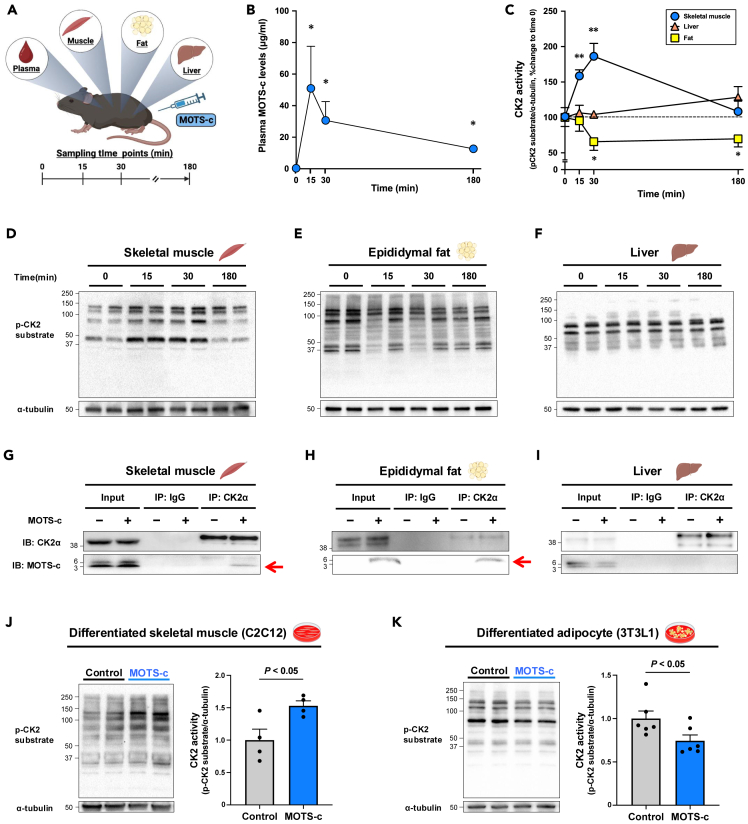
MOTS-c modulates CK2 activity in a tissue-specific manner (A) Experimental design of a single MOTS-c administration (7.5 mg/kg) experiment in young mice (*n* = 5 per time point). (B) Plasma MOTS-c levels after MOTS-c administration. ∗: *p* < 0.05 versus time 0. (C) Quantification of CK2 activity assessed by western blotting in each tissue shown in (E–F). (E–F) CK2 activity assessed by western blotting with p-CK2 substrate antibody in gastrocnemius muscle (D), epididymal fat (E), and liver (F) after MOTS-c administration. ∗*p* < 0.05, ∗∗*p* < 0.01 versus time 0 in same tissue. (G–I) MOTS-c detection following CK2α immunoprecipitation (IP) in gastrocnemius muscle (G), epididymal fat (H), and liver (I) 30 min after MOTS-c administration (7.5 mg/kg). The "-" indicates tissues from non-MOTS-c administered mice, while the "+" indicates tissues from MOTS-c administered mice. (J–K) CK2 activity after 10 min MOTS-c treatment (10 μM) in differentiated skeletal muscle (J) and adipocyte (K). Data are represented as mean ± SEM for (B, C, J, and K).

Then, we next examined the baseline expression levels of CK2α, CK2β, and CK2 activity in various tissues *in vivo*. CK2α expression levels and CK2 activity were higher in liver than those in epididymal fat and skeletal muscle, whereas CK2β expression levels were lower in skeletal muscle than those in liver and epididymal fat (Figures S6A–S6D). These results suggest that the role and response of CK2 to MOTS-c may differ among tissues. Then, we examined the effect of MOTS-c administration on CK2 activity assessed by endogenous proteins containing a CK2 phosphorylation consensus sequence in those tissues (Figure 3A). MOTS-c administration increased MOTS-c levels in plasma (Figure 3B). As we expected, MOTS-c significantly increased CK2 activity by directly binding to CK2α in the skeletal muscle (Figures 3C, 3D, and 3G) without changing CK2α and CK2β expression levels (Figure S7). However, surprisingly, MOTS-c significantly decreased CK2 activity assessed by endogenous proteins containing a CK2 phosphorylation consensus sequence by directly binding to CK2α in epididymal fat (Figures 3C, 3E, and 3H). On the other hand, we did not observe differences in CK2 activity at any time points and the binding between MOTS-c and CK2α in the liver (Figures 3C, 3F, and 3I). The co-immunoprecipitation data are not ideal because these is a non-specific band at 3 kDa in muscle and liver input samples. However, the signal intensity in the MOTS-c administered skeletal muscle input sample is 30% higher than the control (data not shown), suggesting that the band at 3 kDa contains both synthetic MOTS-c and non-specific proteins. Furthermore, since there are clear differences in IP-CK2α samples from muscle and epididymal fat, MOTS-c could bind to CK2α in skeletal muscle and epididymal fat, but not in liver. To confirm the observation in epididymal fat, we examined the effect of 8-week MOTS-c administration on CK2 activity in HFD-fed mouse fat. MOTS-c administration significantly lowered CK2 activity in epididymal fat without changing CK2α and CK2β expression levels (Figures S8A–S8D). Since CK2 inhibition promotes UCP1-dependent beige adipocyte biogenesis,^29^ we also examined UCP1 gene expression levels and found an upregulated *UCP1* expression in the MOTS-c-treated mouse epididymal fat (Figure S8E). Then, we tested the effect of MOTS-c in differentiated mouse skeletal muscle (C2C12) and adipocyte (3T3L1) cells. Supporting the results from the mouse experiments, MOTS-c treatment increased CK2 activity in C2C12 cells, whereas it decreased it in 3T3L1 cells (Figures 3J and 3K). Therefore, although MOTS-c binds to CK2α in both muscle and epididymal fat, the effect of MOTS-s on CK2 activity is different in muscle and fat: MOTS-c phosphorylates CK2 substrates in skeletal muscle, whereas dephosphorylates them in adipose tissue.

### MOTS-c modulates CK2 interactome in tissue-specific manner

**Figure 4:**
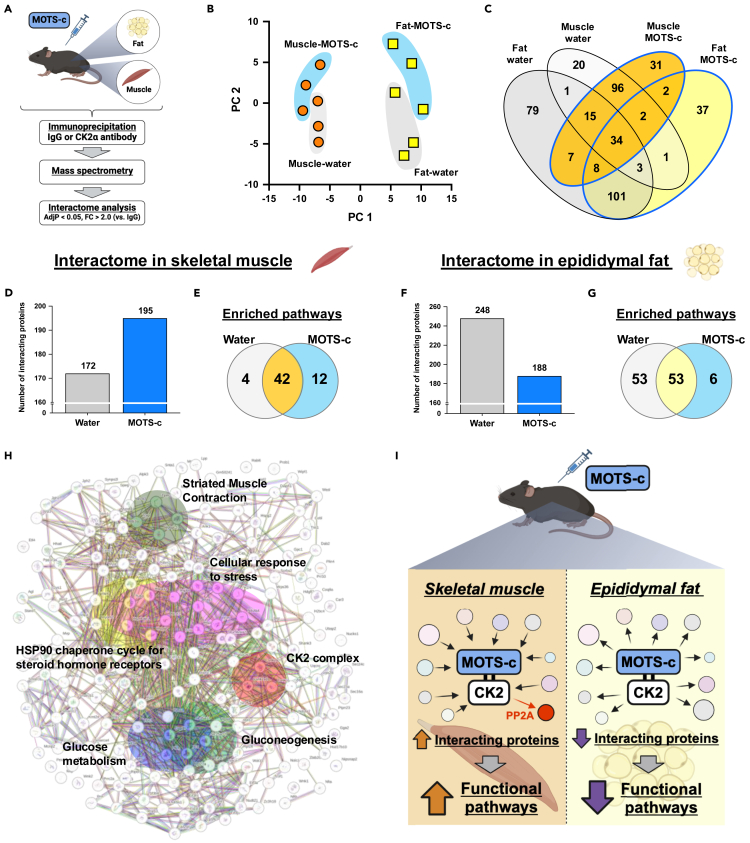
MOTS-c modulates the CK2 interactome in a tissue-specific manner (A) Experimental design for the interactome analyses in young mice (*n* = 3 per condition). Proteome analysis was performed following CK2α immunoprecipitation in gastrocnemius muscle and epididymal fat with/without MOTS-c administration. (B) Principal components (PCs) of control and MOTS-c-treated mouse gastrocnemius muscle and epididymal fat. (C) Venn diagram of interacting proteins in each condition. (D–E) Numbers of CK2 interacting proteins (D) and enriched Reactome pathways (E) in gastrocnemius muscle. (F–G) Numbers of CK2 interacting proteins (F) and enriched Reactome pathways (G) in epididymal fat. (H) Interacting proteins and significantly enriched Reactome pathways in MOTS-c-administered gastrocnemius muscle compared to the control group. The protein-protein interactions and enrichment analysis were assessed by using the STRING database. (I) Summary of the interactome analysis in gastrocnemius muscle and epididymal fat.

To further examine the tissue-specific MOTS-c actions, we assessed the proteins that bind to CK2 by using proteomics analysis following CK2α immunoprecipitation in the skeletal muscle and epididymal fat with/without a single MOTS-c administration (Figure 4A). Principal-component analysis showed a clear separation between muscle and fat, suggesting that the proteins that bind to CK2α were distinctly different between muscle and fat (Figure 4B). Additionally, MOTS-c administration modified those binding proteins in both muscle and fat (Figure 4B). Thirty-four common proteins were identified in all groups, including components of the CK2 complex (i.e., CK2α, CK2α′, and CK2β), whereas other proteins were uniquely detected in tissue- and treatment-specific manner (Figure 4C) (Tables S1, S2, S3, and S4). Interestingly, similarly to the CK2 activity (Figure 3C), in muscle, MOTS-c increased the numbers of binding proteins to CK2 and built new functional pathways, including striated muscle contraction, glucose metabolism, gluconeogenesis, glucose metabolism, cellular response to stress, and the HSP90 chaperone cycle for steroid hormone receptors (Figures 4D, 4E, and 4H; Tables S5 and S6). On the other hand, MOTS-c resulted in a decrease in the numbers of proteins bound to CK2 and their related functional pathways in epididymal fat (Figures 4F and 4G; Tables S7 and S8). The most up- and down-regulated proteins in the MOTS-c-administered muscle compared to water-administered muscle were tropomyosin 3 (TPM3) and ubiquinol-cytochrome c reductase core protein 1 (UQCRC1) (Table S9), respectively, whereas most up- and down-regulated proteins in fat were synaptojanin 2 (SYNJ2) and nuclear casein kinase and cyclin-dependent kinase substrate 1 (NUCKS1), respectively (Table S10). Importantly, MOTS-c also modulated proteins that directly bind to CK2 in both muscle and fat (Figures 4H–4K). In muscle, MOTS-c eliminated Ppp2r2a (PP2A), a protein phosphatase that dephosphorylates AKT, from CK2α and recruited Hsp90ab1 and 8030462N17Rik to CK2α and CK2α′, respectively (Figure S9A). On the other hand, MOTS-c eliminated GAPDH from CK2α′ and recruited 8030462N17Rik to CK2α’ (Figure S9B). Therefore, MOTS-c potently modifies the CK2 interactome in a tissue-specific manner: MOTS-c increasing interacting proteins in skeletal muscle, while decreasing them in epididymal fat (Figure 4I).

### A naturally occurring K14Q MOTS-c is a bio-inactive form of MOTS-c due to its low binding affinity to CK2α

**Figure 5:**
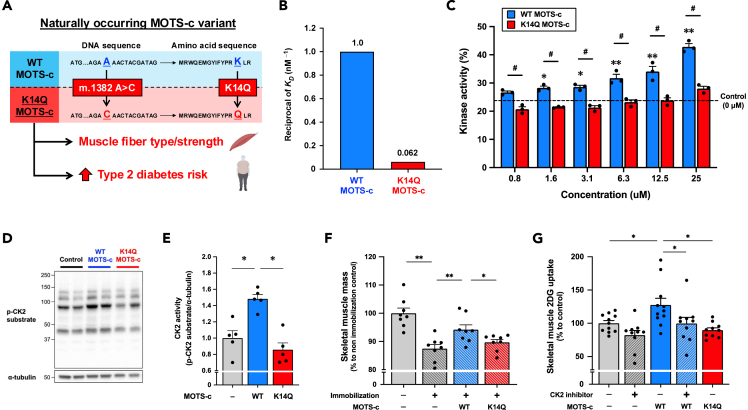
A naturally occurring K14Q MOTS-c is a bio-inactive form of MOTS-c due to its reduced binding to CK2 alpha (A) Nucleotide and amino acid substitutions of naturally occurring MOTS-c variant K14Q MOTS-c, which modulates skeletal muscle function and increases type 2 diabetes risk. (B) Comparison of reciprocal *K*_*D*_ between WT and K14Q MOTS-c assessed by the surface plasmon resonance (Biacore) assay. (C) Comparison of CK2 activating effects between WT MOTS-c and K14Q MOTS-c in cell-free condition (*n* = 3 per group). MOTS-c concentrations are 0, 0.8, 1.6, 3.1, 6.3, 12.5, and 25 μM ∗*p* < 0.05, ∗∗*p* < 0.01 versus control. ^#^*p* < 0.05 versus same concentration of K14Q MOTS-c. (D–E) Comparison of gastrocnemius muscle CK2 activity between WT MOTS-c- and K14Q MOTS-c-administered young mice (2.5 mg/kg, *n* = 5 per group). ∗*p* < 0.05 versus control group. (F) Protective effect of MOTS-c administration (WT or K14Q MOTS-c, 15 mg/kg/day, IP injection) against 8 days of immobilization-induced skeletal muscle atrophy (*n* = 8 per group). Skeletal muscle mass was assessed by a total mass of gastrocnemius, plantaris, and soleus muscles. ∗*p* < 0.05, ∗∗*p* < 0.01. (G)Skeletal muscle 2-deoxy-d-glucose (2DG) uptake after MOTS-c administration (WT or K14Q, 7.5 mg/kg) with/without CK2 inhibitor (CX-4945, 25 mg/kg) (*n* = 10–11 per group). ∗*p* < 0.05. Data are represented as mean ± SEM for (C, E, F, and G).

Within the MOTS-c coding region there is a naturally occurring, single nucleotide polymorphism (SNP) (m.1382A>C, rs111033358), causing a K14Q amino acid substitution of MOTS-c, and this variant increases the risk of T2D^11^ as well as modulates skeletal muscle property and function^14^ (Figure 5A). Additionally, although WT MOTS-c improves glucose metabolism in high-fat-diet-fed mice,^1^^,^^11^ the K14Q MOTS-c variant peptide does not improve it,^11^ suggesting that K14Q MOTS-c is a bio-inactive form of MOTS-c. Thus, we examined whether CK2-binding is involved in these different biological effects of WT MOTS-c and K14Q MOTS-c. A surface plasmon resonance assay demonstrated that the dissociation constant (*K*_*D*_) of K14Q MOTS-c and CK2α was 16.2 nM (Figure 10A), showing that the binding affinity of K14Q MOTS-c to CK2α was more than an order-of-magnitude weaker than that of WT MOTS-c (Figure 5B). Next, we compared the effect of WT and K14Q MOTS-c on the ability of CK2 to phosphorylate its substrate by using a cell-free kinase activity assay. We observed that K14Q MOTS-c did not increase CK2 activity in cell-free system (Figure 5C). Since we previously reported that WT MOTS-c increased CK2 activity and phospho-AKT Ser473 in MOTS-c-administered mouse skeletal muscle,^10^^,^^12^ we assessed them in WT and K14Q MOTS-c-administered mouse skeletal muscle. Supporting the kinase activity assay, while WT MOTS-c increased CK2 activity and AKT phosphorylation at Ser473 in mouse skeletal muscle, K14Q MOTS-c did not increase them (Figures 5D and 5E; Figures S10B and S10C). These data suggest that the naturally occurring K14Q MOTS-c is a bio-inactive form of MOTS-c due to its low binding affinity to CK2α.

### CK2 activation has a crucial role in MOTS-c-induced improvements in muscle mass and glucose uptake

Next, we compared the effects of WT MOTS-c and K14Q MOTS-c on muscle functions *in vivo*. Given that MOTS-c prevents muscle wasting in high-fat-diet-fed and immobilized mice,^10^^,^^12^ we compared the protective effects of WT and K14Q MOTS-c against immobilization-induced muscle atrophy. As we expected, WT MOTS-c attenuated immobilization-induced skeletal muscle atrophy, whereas K14Q MOTS-c failed to attenuate it (Figure 5F). An unbiased RNA sequencing and its downstream enrichment analysis suggested that differentially expressed genes between WT and K14Q MOTS-c-treated mouse gastrocnemius muscles were associated with multiple muscle-related pathways, including PI3K-AKT signaling pathway (Figures S11A and S11B; Table S11). We further examined if CK2 activation is involved in MOTS-c-induced muscle glucose uptake.^1^ Administration of WT MOTS-c significantly increased 2-deoxy-d-glucose (2DG) uptake into the skeletal muscle, but K14Q MOTS-c did not increase it (Figure 5G). Inhibiting CK2 activity by its specific inhibitor CX-4945 led to decreased muscle glucose uptake (*p* = 0.053), whereas co-administration of CK2 inhibitor with MOTS-c prevented the increase in muscle glucose uptake by MOTS-c (Figure 5G). To confirm the involvement of CK2 in MOTS-c-induced muscle glucose uptake, we knocked down (KD) CK2α, a direct binding protein of MOTS-c, by using an antisense oligonucleotide (ASO) in differentiated human skeletal muscle cells. Although WT MOTS-c increased muscle 2DG uptake in the scramble ASO group, it did not increase it in the CK2α KD muscle cells (Figures S12A and S12B). The bio-inactive K14Q MOTS-c did not increase muscle 2DG uptake in either scramble ASO or CK2α KD groups (Figure S12B). These data demonstrate that MOTS-c modulates skeletal muscle functions, such as muscle mass and glucose uptake, through activating CK2.

### The bio-inactive K14Q MOTS-c increases risks of sarcopenia and type 2 diabetes in humans

**Figure 6:**
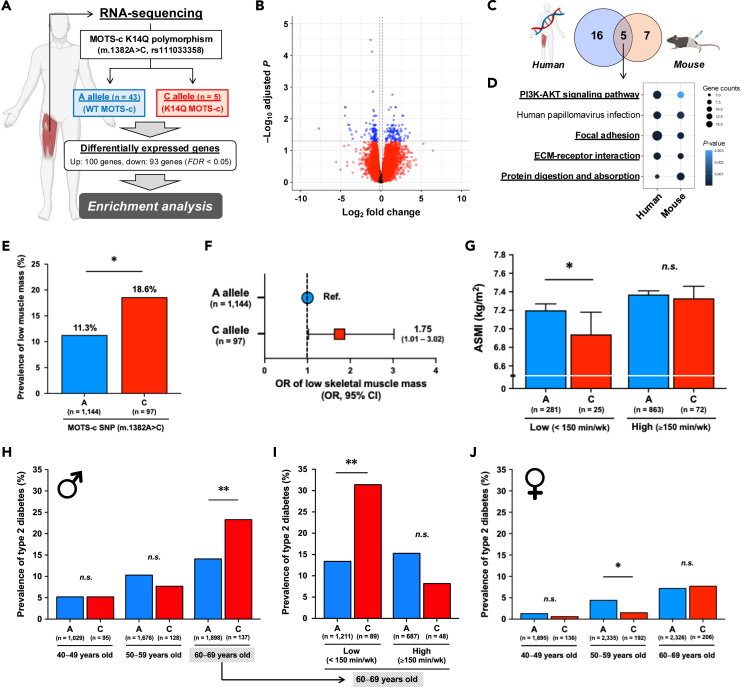
The bio-inactive MOTS-c variant increases risks of sarcopenia and type 2 diabetes in an age- and physical-activity-dependent manner (A) A schema of the RNA sequencing analysis in human skeletal muscle from the A allele and C allele carriers of MOTS-c single nucleotide polymorphism (SNP) (m.1382A>C, rs111033358) in 48 Japanese males and females. (B) Differentially regulated genes in human skeletal muscle between the A and C allele carriers. Age and sex were used as covariates. (C–D) The overlapped enriched pathways between human skeletal muscles from the A or C allele carriers and skeletal muscles from WT or K14Q MOTS-c-treated immobilized mice. KEGG pathways were used for the enrichment analyses. (E) Percentage of people with low skeletal muscle mass between the A and C allele carriers of MOTS-c SNP (m.1382A>C, rs111033358) in 1,241 Japanese individuals. Skeletal muscle mass was measured by dual-energy X-ray absorptiometry, and appendicular skeletal muscle mass index (ASMI) was calculated. The cutoff values for low skeletal muscle mass are ASMI <7.0 kg/m^2^ for men and <5.4 kg/m^2^ for women. (F) Odds ratio (OR) and 95% confidence interval of low skeletal muscle mass between the A allele and C allele carriers of MOTS-c SNP (m.1382A>C, rs111033358). OR was adjusted for age and sex. (G) Skeletal muscle mass assessed by ASMI between the A and C allele carriers of MOTS-c SNP (m.1382A>C, rs111033358) in low and high physical activity groups. A cutoff value for physical activity is 150 min of moderate-to-vigorous physical activity (MVPA) per week base on the World Health Organization guideline. Data are represented as mean ± SEM. (H) The prevalence of type 2 diabetes in the A and C allele carriers of MOTS-c SNP (m.1382A>C, rs111033358) in 4,966 Japanese males with considering age. (I) The prevalence of type 2 diabetes in the A and C allele carriers in subjects in the 60s with considering physical activity levels. A cutoff value of the physical activity is 150 min of MVPA per week base on the World Health Organization guideline. (J) The prevalence of type 2 diabetes in the A and C allele carriers of MOTS-c SNP (m.1382A>C, rs111033358) in 6,890 Japanese females with considering age.∗*p* < 0.05, ∗∗*p* < 0.01.

To examine the influence of the bio-inactive K14Q MOTS-c in human subjects, we first compared skeletal muscle gene expression pattern between the A allele (WT MOTS-c) and C allele (K14Q MOTS-c) carriers of the m.1382A>C variant (Figure 6A;Table S13). We detected a total of 193 differentially expressed genes (*FDR* < 0.05, Table S14), and these genes were associated with multiple muscle-related pathways, including the PI3K-AKT signaling pathway (Table S15). Interestingly, the overlapped pathways between human subjects with A or C alleles and mouse treated with WT or K14Q MOTS-c include common muscle related pathways, such as PI3K-AKT signaling pathway, focal adhesion, ECM-receptor interaction, and protein digestion and absorption (Figures 6C–6D). These observations suggest that the m.1382A>C polymorphism significantly regulates skeletal muscle gene expressions and could influence skeletal muscle phenotypes.

Since MOTS-c prevents skeletal muscle wasting induced by immobilization (Figure 5F) as well as HFD,^10^ we further examined the association between the m.1382A>C and skeletal muscle mass in 1,241 Japanese population (Table S16). The C allele carriers exhibited a higher prevalence of low skeletal muscle mass, a risk of sarcopenia, assessed by appendicular skeletal muscle mass index (ASMI) and its cutoff values (11.3% versus 18.6%, *p* < 0.05) (Figure 6E; Table S16). This significant association remained significant after correcting for age and sex (odds ratio: 1.75, 95% confidence interval: 1.01–3.02) (Figure 6F; Table S17). Thus, a deficiency of functional MOTS-c increases the risk of sarcopenia, highlighting the importance of this mitochondrial microprotein in aging-related frailty. Since we have previously demonstrated that high levels of physical activity can counteract the negative effect of functional MOTS-c deficiency on T2D risk^11^ and since exercise increases muscle CK2 activity (Figures 1D and 1E), we examined the interaction between MOTS-c SNP and physical activity levels on muscle mass in humans (Figure 6G; Table S18). We used 150 min of moderate to vigorous physical activity (MVPA) per week as a cutoff value for high versus low physical activity based on the World Health Organization guidelines. As expected, the C allele carriers in the low physical activity group exhibited a significantly lower appendicular skeletal muscle mass index (ASMI) compared to the A allele carriers, but there was no difference in the high physical activity group (Figure 6G), further demonstrating that exercise can compensate for the functional MOTS-c deficiency. Beyond a sedentary lifestyle, aging decreases muscle CK2 activity (Figures 1A and 1B); we next examined the interaction between the MOTS-c SNP status and aging on T2D incidence. Although the prevalence of T2D was not different between the groups in the 40s and 50s age ranges, the C allele carriers exhibited a significantly higher risk of T2D in their 60s (Figure 6H; Table S19). The association between MOTS-c SNP and T2D was confirmed by multiple regression analysis with age, sex, and/or physical activity levels as covariates (Table S20). Additionally, the C allele carriers in their 60s also showed a significantly lower skeletal muscle mass index and a trend to have lower grip strength in males (Figures S13A and S13B; Table S21), but not in females (Figures S13C–S13D; Table S21). Those observations suggest that aging accelerates the negative effect of MOTS-c deficiency by synergistically decreasing muscle CK2 activity in males. Then, we examined if physical activity could counteract the increased T2D risk associated with aging and functional MOTS-c deficiency. As expected, although the C allele carriers exhibited a significantly higher prevalence of T2D in the low physical activity group, there was no difference in the high physical activity group in males (Figure 6I; Table S22). On the other hand, in females, the C allele carriers exhibited a lower risk of T2D in their 50s (Figure 6J; Table S19), suggesting that there might be a sex difference in the response to the MOTS-c variant. Altogether, these results suggest that the naturally occurring functional MOTS-c deficiency increases the risk of sarcopenia and T2D by interacting with age and physical activity levels, and high physical activity levels, ≥150 min of MVPA per week, can alleviate the increased risk of sarcopenia and T2D in elderly men with MOTS-c deficiency by activating muscle CK2. However, the effect of the MOTS-c variant might be differentially influenced by age and sex and requires further investigations.

## Discussion

CK2 is a ubiquitous and highly conserved serine-threonine kinase that regulates several cellular functions, including glucose metabolism.^16^ More specifically, CK2 inhibition suppresses glucose uptake by reducing AKT signaling in adipose tissue^17^ and other cells,^18^ demonstrating that high CK2 activity promotes glucose uptake. In this study, we observed that CK2 inhibition and CK2α KD decreased muscle glucose uptake, suggesting that CK2 activity has a crucial role in regulating glucose metabolism in skeletal muscle as well. On the other hand, MOTS-c administration increased muscle glucose uptake, which was blocked by CK2 inhibition and CK2α KD. Thus, MOTS-c administration increases muscle glucose uptake by interacting with CK2α and activating CK2. Since we observed strong direct binding between MOTS-c and CK2α *in vitro* and *in vivo*, this study demonstrated that CK2 is a direct and functional target of MOTS-c and that the MOTS-c/CK2 pathway has crucial roles in muscle glucose metabolism.

MOTS-c administration modified the CK2 interactome in a tissue-dependent manner. PP2A, a protein phosphatase that dephosphorylates AKT, was displaced from the CK2 complex in the muscle in response to MOTS-c. We previously demonstrated that MOTS-c treatment increased phospho-AKT Ser473 expression in skeletal muscle.^1^^,^^10^ Thus, dissociating PP2A from the complex may explain the underlying mechanism of how MOTS-c phosphorylates AKT in muscle. On the other hand, MOTS-c administration recruited HSP19AB1 into the CK2 complex and modified this chaperone network in skeletal muscle. Since chaperones have been recognized to involved protein folding and as protein misfolding is suggested to cause muscle dysfunction,^30^ MOTS-c administration could prevent muscle dysfunction by rejuvenating this chaperone network. Our previous studies also demonstrated that MOTS-c modulated expression levels of heat shock proteins, further modulating protein folding under glucose restriction and serum deprivation in skeletal muscle.^6^ Thus, MOTS-c is emerging as a regulator of protein folding in skeletal muscle as part of its metabolic effects.

Proteomics analysis following CK2α immunoprecipitation demonstrated that MOTS-c administration modulated proteins bound to the CK2 complex. In skeletal muscle, TPM3 (tropomyosin 3), a component of the sarcomeric thin filament troponin-tropomyosin complex involved in muscle contraction, showed the biggest increase in the MOTS-*c*-treated mice compared to controls. TPM3 has been linked to myopathy,^31^^,^^32^ and a dominant negative mutation of TPM3 causes muscle weakness.^33^ On the other hand, UQCRC1 (ubiquinol-cytochrome c reductase core protein I) showed the biggest reduction in MOTS-c-treated muscle. It has been demonstrated that increased UQCRC1 expression causes skeletal muscle lipid accumulation.^34^ Since MOTS-c increases fatty acid utilization^1^ and prevents skeletal muscle atrophy by suppressing fatty infiltration,^12^ it is possible that MOTS-c prevented lipid accumulation through interacting with UQCRC1. Therefore, MOTS-c appears to prevent skeletal muscle dysfunction by modulating CK2 interactome in specific ways.

In contrast to skeletal muscle, MOTS-c administration decreased CK2 activity in fat. It has been demonstrated that CK2 inhibits brown fat formation and inhibition of CK2 promotes beige adipocyte biogenesis.^29^^,^^35^ In this study, both acute and chronic MOTS-c administration decreased CK2 activity in fat, and 8 weeks of MOTS-c treatment significantly increased UCP1 mRNA expression in fat, suggesting that chronic MOTS-c treatment leads to beige adipocyte biogenesis. In fact, Lu et al. have demonstrated that MOTS-c administration increased white-fat browning and thermogenesis in cold exposure and ovariectomized (OVX) mice.^36^^,^^37^ Therefore, MOTS-c may mediate some of its metabolic effects by increasing beige-adipocyte biogenesis and thermogenesis through the inhibition of CK2 activity in fat.

The interactome analyses revealed that the MOTS-c/CK2 complex interacts with numerous proteins in muscle and fat tissues, some of which may exert adverse effects. However, the analyses did not clarify whether these interactions involve phosphorylation or dephosphorylation. Consequently, it remains unclear whether MOTS-c exerts adverse effects in these tissues. Further detailed analyses are needed to elucidate the nature and direction of these interactions.

Although the differences in skeletal muscle mass and grip strength between A and C allele carriers are relatively small, we believe they are physiologically important for several reasons. Skeletal muscle mass is suggested to decrease by 1%–2% per year,^38^ making the ∼7% difference observed in our study significant, as it may equate to muscle loss over 3–7 years. Additionally, the difference of 1 kg in grip strength between allele carriers could be meaningful, given that a 5 kg reduction in grip strength is associated with a 1.16 hazard ratio for all-cause mortality.^39^ Finally, considering that these differences are caused by a single nucleotide polymorphism, the small effect size is reasonable because these phenotypes are not genetic diseases.

In summary, we identified CK2 as a direct and functional binding partner of MOTS-c and showed that MOTS-c modulated CK2 activity in a tissue-specific manner. MOTS-c prevented immobilization-induced muscle atrophy and increased muscle glucose uptake by activating CK2 while suppressing CK2 activity in fat. A naturally occurring K14Q MOTS-c was shown to be a bioinactive form of MOTS-c due to its reduced binding to CK2, and carriers of this mutation are at a higher risk of developing sarcopenia and T2D in an age- and physical-activity-dependent fashion in males. The effect of the MOTS-c variant might be differentially influenced by age and sex and requires further investigations. Altogether, our findings provide strong evidence that CK2 is a direct and functional binding partner of MOTS-c and that MOTS-c regulates CK2 activity in a tissue-specific manner. These results may lead to new strategies for preventing sarcopenia and T2D.

### Limitation of the study

Microprotein detection is one of the most challenging techniques because of its size. MOTS-c is not an exception, and MOTS-c detection by western blot using an MOTS-c antibody is still challenging. Although MOTS-c should localize at 2.2 kDa based on the calculated molecular weight, we previously demonstrated that a band around 6 kDa (between 6 and 14 kDa) is endogenous MOTS-c and a band around 3 kDa is non-specific by peptide blocking approach in C2C12 myotube.^12^ This is potentially because of oligomerization or post-translational modification. On the other hand, we found that synthetic MOTS-c by a single administration localized around 3 kDa in skeletal muscle and 6 kDa in epididymal fat. Thus, the localization could be different between endogenous and exogenous MOTS-c as well as tissue types. Further study is necessary to understand about microprotein detection well.

## Resource availability

### Lead contact

Further information and requests for resources and reagent should be directed to and will be fulfilled by the lead contact, Pinchas Cohen (hassy@usc.edu).

### Materials availability

This study did not generate new unique reagents.

### Data and code availability

#### Data

The raw RNA sequencing data in humans and mice are deposited in the GEO database, GSE274907 and GSE274924, respectively. The raw proteome data in mouse skeletal muscles are deposited in theMassIVE database, MSV000096057.

#### Code

The original code has been deposited at the GitHub (https://github.com/hiroshi-kumagai/M3-1382AC).

#### All other items

Any additional information required to reanalyze the data reported in this paper is available from the lead contact upon request.

## Acknowledgments

This study was supported by 10.13039/100000002NIH grants R01AG069698, RF1AG061834, R01AG068405, P30AG068345, P01AG055369, R01CA231219, and R01HL158691. The J-MICC Study in Saga region was supported by Grants-in-Aid for Scientific Research for Priority Areas of Cancer (No. 17015018) and Innovative Areas (No. 221S0001) and by the 10.13039/501100001691Japan Society for the Promotion of Science KAKENHI Grant (Nos. 16H06277, 22H04923 [CoBiA], 18390182, and 20249038) from the Japanese Ministry of Education, Culture, Sports, Science and Technology. The WASEDA’S Health Study was supported by the 10.13039/501100001691Japan Society for the Promotion of Science KAKENHI Grant (Nos. 17K13241, 18K17982, 19H04065, 21K17693) and the MEXT-Supported Program for the Strategic Research Foundation at Private Universities (No. S1511017) from the Ministry of Education, Culture, Sports, Science and Technology. The authors thank Dr. Shuxing Li at the Center of Excellence in NanoBiophysics in USC for his technical assistance with the Surface plasmon resonance (Biacore) assay. We thank all the staff and participants in the J-MICC Study and WASEDA’S Health Study.

## Author contributions

H.K., S.-J.K., B.M., K.Y., and P.C. conceptualized the study. H.K., S.-J.K., B.M., N.L., R.R., H.M., K.C., and K.Y. planned and performed *in vitro* experiments. H.K., S.-J.K., B.M., S.L., J.A.W., J.S., and K.Y. planned and performed mass spectrometry experiments. H.K., S.-J.K., B.M, S.L., J.W., T.N., H.M., S.D., and K.Y. planned and performed *in vivo* experiments. H.K., B.M., and M.E.K. performed bioinformatics methodologies and visualization. H.Z., K.T., TJ.O., Y.N., N.F., E.M.-M., M.T., M.F., Y.T., H.N., R.K., S.T., T.M., K.O., M.H., C.I., Y.Y., Y.H., and K.T. planned and performed, analyzed, and/or interpreted human studies. H.K. H.M., K.Y., and P.C. wrote the original draft. All authors confirmed and approved the final version of the manuscript.

## Declaration of interests

Pinchas Cohen is an advisor to and stockholder in CohBar Inc. UCLA has licensed the intellectual property on MOTS-c, on which Pinchas Cohen is listed as an inventor, to CohBar. None of the other authors has any conflicts of interest, financial or otherwise, to disclose.

## STAR★Methods

### Key resources table

**Table:** 

REAGENT or RESOURCE	SOURCE	IDENTIFIER
**Antibodies**		

Phospho-CK2 Substrate antibody	Cell Signaling Technology	8738; RRID: AB_2797653
Custom MOTS-c antibody	YenZym	–
Phospho-Akt (Ser473) antibody	Cell Signaling Technology	4060; RRID: AB_2315049
Anti-AKT1 (phospho S129) antibody	abcam	ab133458
Akt antibody	Cell Signaling Technology	4691; RRID: AB_915783
CK2α antibody	Cell Signaling Technology	2656; RRID: AB_2236816
CK2α antibody (for IP)	Bethyl Laboratories	A300-198A; RRID: AB_185571
Casein kinase IIα Antibody (1AD9) HRP	Santa Cruz Biotechnology	sc-12738 HRP; RRID: AB_2276843
CK2β antibody	Invitrogen	PA5-27416; RRID: AB_2544892
Phospho-CDC37 (Ser13) antibody	Cell Signaling Technology	13248; RRID: AB_2783724
CDC37 antibody	Cell Signaling Technology	4793; RRID: AB_10695539
α-Tubulin Antibody	Cell Signaling Technology	2144; RRID: AB_2210548
Anti-rabbit IgG, HRP-linked Antibody	Cell Signaling Technology	7074; RRID: AB_2099233

**Chemicals, peptides, and recombinant proteins**		

WT MOTS-c	GenScript	–
K14Q MOTS-c	GenScript	–
Casein Kinase II	NEB	P6010S
CK2α	GenScript	–
CK2β	IBL	IBATGP4047
CK2 substrate peptide	Millipore Sigma	12–300
Insulin	Sigma-Aldrich	11070-73-8
ATP	NEB	P0756S
Dexamethasone	Millipore Sigma	265005
3-Isobutyl-1-methylxanthine	Millipore Sigma	I5879
Rosiglitazone	Millipore Sigma	R2408
2-Deoxy-D-glucose	Millipore Sigma	D6134

**Critical commercial assays**		

Kinase-Glo® Luminescent Kinase Assays	Promega	V6711
Glucose Uptake-Glo™ Assay	Promega	J1342
2-Deoxyglucose (2DG) Uptake Measurement Kit	Cosmo Bio	CSR-OKP-PMG-K01H

**Deposited data**		

Human skeletal muscle RNA sequencing data	This study	GSE274907
Mouse skeletal muscle RNA sequencing data	This study	GSE274924
Mouse skeletal muscle interactome (proteome) data	This study	MSV000096057

**Experimental models: Cell lines**		

C2C12	ATCC	CRL-1772™
3T3-L1 MBX	ATCC	CRL-3242
HSMM – Human Skeletal Muscle Myoblasts	Lonza	CC-2580

**Experimental models: Organisms/strains**		

C57BL/6J	Japan SLC, Inc.	–
C57BL/6J	Jackson Laboratory	–
C57BL/6J	NIA Aged Rodent Colony	–

**Oligonucleotides**		

AUM*silence* ASO for CK2α: TAGTCTGTTAACGTCTGGTAC	AUM BioTech	–

**Software and algorithms**		

Image Lab software	Bio-Rad	–
Biacore T200 Evaluation Software	Cytiva Life Science	–
STAR	Dobin et al.^40^	–
GenomicAlignements	Lawrence et al.^41^	–
DESeq2	Love et al.^42^	–
R programming environment (v4.0.3)	The R Project for Statistical Computing	–
STRING	Szklarczyk et al.^43^	–
Metascape	Zhou et al.^44^	–
ColabFold	Mirdita et al.^28^	–
IGV	US San Diego, Broad Institute	–

**Other**		

DMEM, high glucose	Gibco	11965092
Fetal Bovine Serum, USDA Approved, Heat Inactivated	Omega Scientific	FB-02
Horse Serum, New Zealand origin	Gibco	16050122
SkGM™-2 Skeletal Muscle Cell Growth Medium-2	Lonza	CC-3245
DMEM F12, HEPES	Gibco	11330032
DMEM/F12 (Glucose free), with HEPES	Elabscience	PM150323
Rodent Diet With 60 kcal% Fat	Research Diets	D12492
Dynabeads™ Protein G Immunoprecipitation Kit	Invitrogen	10007D
Nitrocellulose/Filter Paper Sandwiches	Bio-Rad	1620213
SuperBlock (PBS) Blocking Buffer	Thermo Scientific	37516
Clarity Western ECL Substrate	Bio-Rad	1705061
Clarity Max Western ECL Substrate	Bio-Rad	1705062
Halt™ Protease and Phosphatase Inhibitor Single-Use Cocktail (100×)	Thermo Scientific	78442
8–16% Mini-PROTEAN® TGX™ Precast Protein Gels, 10-well, 50 μL	Bio-Rad	4561104
4–20% Mini-PROTEAN® TGX™ Precast Protein Gels, 10-well, 50 μL	Bio-Rad	4561094
NuPAGE™ Bis-Tris Mini Protein Gels, 4–12%, 1.0–1.5 mm	Invitrogen	NP0321BOX
NuPAGE™ MOPS SDS Running Buffer (20×)	Invitrogen	NP0001
NuPAGE™ MES SDS Running Buffer (20×)	Invitrogen	NP0002
TRIzol™ Reagent	Invitrogen	15596026
NEBNext® Ultra™ II Directional RNA Library Prep Kit for Illumina®	NEB	E7760
Universal Plus mRNA-Seq with NuQuant®	Tecan Life Sciences	0524
*Quick*-RNA Miniprep Kit	Zymo Research	R1055
Cytiva SERIES S SENSOR CHIP CM5 1/PK	Cytiva	29104988
Trans-Blot Turbo RTA Mini 0.2 μm PVDF Transfer Kit, for 40 blots	Bio-Rad	1704272
Pierce™ BCA Protein Assay Kits	Thermo Scientific	23225

### Experimental model and study participant details

#### Mouse experiments

Male C57BL/6J mice were obtained from the Jackson Laboratory (Bar Harbor, ME), NIA Aged Rodent Colony, or Japan SLC, Inc (Hamamatsu, Japan), and acclimated to the new environment for one week before the experiments. The mice were housed in a controlled environment with a 12-h light-dark cycle and had free access to food and water. All animal procedures were carried out according to the University of Southern California and Juntendo University’s Animal Care and Use Committee and Animal Research.

Exercise training experiment: For the running exercise study, male C57BL/6J mice (10 weeks of age) were assigned to control or exercise groups. Mice in the exercise group were housed in a cage containing a running wheel and performed voluntary wheel running for four weeks. All mice had free access to a running wheel 14.5 cm in diameter with a 5 cm-wide running surface (WW-3202, TOKIWA KAGAKU KIKAI Co., LTD, Tokyo, Japan). At the end of the experiment, mice were euthanized, and gastrocnemius muscle was collected. The collected samples were stored at −80°C for further analyses. We used the entire gastrocnemius muscle for the analysis to avoid potential bias.

Two-month MOTS-c treatment experiment: Male C57BL/6J mice at 10 weeks of age were randomly assigned to one of the experimental groups: high fat diet (HFD, Research Diets, Inc., New Brunswick, NJ) group (sterilized water) or HFD and MOTS-*c*-treated groups (5 mg/kg/day). Sterilized water or MOTS-c was injected twice a day by intraperitoneal injection for two months. At the end of the experiment, the mice were euthanized and liver, epididymal fat, and quadriceps muscle were corrected 3–4 h after the last injection. The collected samples were stored at −80°C for further analyses. The entire gastrocnemius muscle was used for the analysis to avoid potential bias.

Acute MOTS-c administration experiment: Male C57BL/6J mice at 10–12 weeks of age were randomly assigned to one of the experimental time points: 0 min, 15 min, 30 min, or 180 min. MOTS-c (7.5 m/kg) was administered by intraperitoneal injection. At each time point, plasma was collected under anesthesia with isoflurane. Then, the mice were euthanized and liver, epididymal fat, and gastrocnemius muscle were collected. For the coimmunoprecipitation assay, liver, epididymal fat, and gastrocnemius muscle were collected 30 min after the administration. The collected samples were stored at −80°C for further analyses. The entire gastrocnemius muscle for the analysis to avoid potential bias.

Immobilization experiment: To check the preventive effect of MOTS-c on immobilization-induced skeletal muscle atrophy, male C57BL/6J mice at 10 weeks of age were randomly assigned to one of the four experimental groups: non-immobilization control group (twice a day sterilized water injection for eight days), immobilization control group (twice a day sterilized water injection for eight days), immobilization and MOTS-c treated group (15 mg/kg/day MOTS-c injection for 8 days, twice a day IP injection), or immobilization and K14Q MOTS-c treated group (15 mg/kg/day K14Q MOTS-c injection for 8 days, twice a day IP injection). Eight days hindlimb immobilization was performed by using casting material (Scotchcast Plus-J; 3M Health Care, St. Paul, Minnesota). At the time of casting, mice were anesthetized with the inhalant isoflurane and the casting material was attached to both hind legs. Casting material was checked every day for damage and repaired as necessary. At the end of the experiment, mice were euthanized, and gastrocnemius, plantaris, and soleus muscles were collected. The collected samples were stored at −80°C for further analyses. The entire gastrocnemius muscle was used for the analysis to avoid potential bias.

2-deoxyglucose (2-DG) uptake experiment: Male C57BL/6J mice at 14–15 weeks of age were received sham injection (injected nothing) for seven days before the experiment to acclimated to the invasive stimulus by the injection. A selective CK2 inhibitor CX-4945 (MCE) was dissolved in 0.9% saline containing 5% DMSO. After 120 min of fasting, mice were intraperitoneally injected with a CK2 inhibitor at 25 mg/kg or vehicle.^45^ Two hours later, mice were injected insulin at 0.75 U/kg (MilliporeSigma) and MOTS-c at 7.5 mg/kg (GenScript) or sterilized water. Then 10 min later, 5 μg of 2DG (Sigma-Aldrich) was injected.^46^ Mice were euthanized 45 min after 2DG injection and gastrocnemius muscle was collected. The collected samples were stored at −80°C for further analyses. The entire gastrocnemius muscle was used for the analysis to avoid potential bias.

#### Cell culture experiments

Mouse skeletal muscle myoblast cells (C2C12, ATCC), mouse fibroblast cells (3T3-L1 MBX, ATCC), and human skeletal muscle myoblast cells (HSMM, Lonza) were purchased and used for the experiments.

C2C12 cells were purchased from ATCC and cultured in DMEM with 10% FBS (Omega Scientific) at 37°C in 5% CO2. To differentiate C2C12 into myotubes, the media was replaced with DMEM with 2% horse serum (Gibco) every 48 h for 6 days until the cells were fully differentiated. Then, the media was replaced with DMEM without serum and incubated for 24 h. To investigate the effect of MOTS-c treatment on CK2 activity, the differentiated myotubes were incubated with or without 10 μM MOTS-c (Genscript) for 10 min.

3T3-L1 cells were purchased from ATCC (Cat No. CRL-3242) and cultured in DMEM +10% FBS (Omega Scientific) at 37°C in 5% CO2. To differentiate 3T3-L1 pre-adipocytes, pre-adipocyte expansion medium was replaced with differentiation medium (DMEM, 10% FBS, 1 μM Dexamethasone, 0.5 mM IBMX, 1 μg/mL insulin, 2.0 μM Rosiglitazone) for 48 h, then replaced with adipocyte maintenance medium (DMEM 10% FBS, 1 μg/mL insulin) every 2 days for 7 days. Then, the media was replaced with DMEM without serum and incubated for 24 h. To investigate the effect of MOTS-c treatment on CK2 activity, the differentiated adipocytes were incubated with or without 10 μM MOTS-c (Genscript) for 10 min.

HSMM cells were purchased from Lonza and cultured in SkGM-2 Skeletal Muscle Cell Growth Medium-2 (Lonza) at 37°C in 5% CO2. The donor of the HSMM cells was a 40 years old healthy male. To differentiate HSMM into myotubes, the media was replaced with DMEM F12 (Gibco, Cat No. 11330) with 2% horse serum (Gibco) every 48 h for 4 days until the cells were fully differentiated. The fully differentiated myotubes were incubated with AUM*silence* ASO targeting CK2α (5 μM, 5’ – 3’: TAGTCTGTTAACGTCTGGTAC) or AUM*scr* ASO for scramble control (5 μM) in DMEM F12 (Gibco) without serum for 24 h to knockdown CK2α. Since the AUM*silence* ASOs are self-deliverable and do not need any transfection reagents, they can minimize the toxicity to cells. On the day of the glucose uptake assay, the media were replaced with glucose free DMEM F12 (Elabscience) without serum. The media contained 1 μM of insulin (MilliporeSigma) and 10 μM of WT or K14Q MOTS-c (GenScript). Then, the treated cells were used for 2-deoxyglucose uptake (2-DG) assay.

#### Human experiments

The information about the following human studies is summarized in Table S12.

##### Skeletal muscle gene expression cohort

A total of 48 healthy Japanese individuals (23, male; 25 female) were recruited to the muscle gene expression cohort. The characteristics of the participants in the muscle gene expression cohort are shown in (Tables S12, S13, S14, and S15). The participants were instructed to avoid strenuous exercise for 24 h prior to biopsy. After overnight fasting for >12 h, muscle biopsies were taken from the vastus lateralis using a 14 G biopsy needle (Bard Monopty Max Core, C. R. Bard, NJ, USA) under sterile conditions and local anesthesia (1% lidocaine). The biopsies were collected using ultrasound imaging (Noblus, Aloka, Tokyo, Japan) guidance from the dominant leg of each participant approximately 15 cm proximal from the lateral knee joint space and 2 cm deep from the fascia. The samples were cleaned quickly on ice to remove any visible non-muscle material and frozen immediately in liquid nitrogen. The muscle samples were stored at −80°C until needed for further analyses. The study protocol was approved by the Ethics Committee of Juntendo University.

##### WASEDA’S Health Study

The association of the MOTS-c K14Q polymorphism with skeletal muscle mass was examined using baseline survey data from Waseda Alumni’s Sports, Exercise, Daily Activity, Sedentariness, and Health (WASEDA’S Health) Study, which is a prospective cohort study among the alumni of Waseda University and their spouses aged ≥40 years.^47^^,^^48^^,^^49^^,^^50^ The WASEDA’S Health Study consists of four cohorts (cohorts A–D) with different measurement items, and the participants selected one of the four cohorts when registering for the study. The present study comprised a total of 1387 individuals (men: *n* = 916; women: *n* = 471) who participated in the baseline survey of cohort D between March 2015 and February 2020. Of the 1387 individuals, we excluded participants who met the following criteria: 1) lack of body composition data measured by dual-energy X-ray absorptiometry (DXA; *n* = 67); 2) incomplete or invalid questionnaire (*n* = 63); 3) lack of blood sample for DNA extraction (*n* = 8); 4) not passing quality control of genotype data (*n* = 8). Based on the above criteria, 1241 Japanese adults (men, *n* = 827; women, *n* = 414) were included in the analysis (Tables S12 and S16–S18). All participants provided written informed consent before enrolling in the study, which was approved by the Ethical Review Committee of Waseda University. The study was conducted in accordance with the principles of the Declaration of Helsinki.

DXA scans (Delphi An until December 2016 and Horizon A after January 2017, Hologic Inc., Marlborough, MA, USA) were used to assess body composition as described previously.^47^^,^^48^^,^^49^ DXA-measured appendicular skeletal muscle mass (ASM) and ASM index (ASMI; ASM/height^2^) were used as a skeletal muscle mass index. Low muscle mass was defined based on the AWGS 2019 cutoffs for low muscle mass^51^ as described previously.^47^^,^^48^^,^^49^ We measured hand grip strength using a dynamometer (grip strength: TKK5401, Takei Inc., Niigata, Japan) to assess muscular fitness as described previously.^47^^,^^48^^,^^49^ Moderate to vigorous physical activity (MVPA) was assessed using the Global Physical Activity Questionnaire,^52^ and the time spent in total MVPA (min/week) was calculated. Participants were categorized into high and low MVPA groups according to the WHO physical activity guidelines 2020 (MVPA ≥150 min/week).^53^

Total DNA was extracted from venous blood using QIAamp DNA Blood Midi Kit (Qiagen, Hilden, Germany). DNA quality was evaluated by agarose gel electrophoresis and spectrophotometry. We genotyped approximately 550,000 SNPs including MOTS-c K14Q polymorphism using Infinium HumanCoreExome BeadChip (Illumina, Inc., San Diego, CA, USA) according to the manufacturer’s protocol, followed by genotype calling using the GenTrain clustering algorithm in the GenomeStudio 2.0.5 (Illumina, Inc., San Diego, CA, USA). Quality control of genotype data was performed using PLINK 1.9 to remove the individuals that potentially lead to a bias. We removed the participants with either one of each pair of second-degree relatives defined based on identity-by-descent (PI-HAT >0.1875; *n* = 6), genetic outliers detected by principal component analysis (*n* = 2), individuals with discordance between self-reported sex and genotype-inferred sex (*n* = 1), and missing SNP call for MOTS-c K14Q (*n* = 2). Of the 11 individuals not passing the quality control, 3 individuals had been already excluded according to the criteria as mentioned before; therefore, 8 individuals were excluded through the quality control of genotype data.

##### Japan multi-institutional collaborative (J-MICC) study

The association of the MOTS-c K14Q polymorphism with the prevalence of type 2 diabetes and skeletal muscle mass/strength were examined in the J-MICC study.^54^ This study consisted of 12,068 subjects in Saga City (men, 5,078; women, 6,990) who were between 40 and 69 years old at the baseline. The Saga J-MICC Study was approved by the ethics committees of both the Saga University Faculty of Medicine and Nagoya University Graduate School of Medicine. The study conformed to the principles outlined in the Declaration of Helsinki. Written informed consent was obtained from all subjects before their inclusion in the study.

*J-MICC 1*^*st*^*study*: The baseline survey was conducted from November 1, 2005 through December 22, 2007.^55^ A self-administered questionnaire was used to collect data on smoking, dietary habits, current medication, disease history, and family history. Daily physical activity was objectively measured using an accelerometer (Life-Corder; Suzuken, Nagoya, Japan) as previously described.^56^ Height and weight were measured to the nearest 0.1 cm and 0.1 kg, respectively. Body mass index (BMI) was calculated as the weight in kilograms divided by the square of the height in meters (kg/m2). Waist circumference was measured to the nearest 0.1 cm at the midpoint between the lower costal margin and the iliac crest using a calibrated measuring tape. The HbA1c (%) level was measured and converted from the Japan Diabetes Society (JDS) to the National Glyco-hemoglobin Standardization Program (NGSP) by using the following equation published by the JDS: NGSP (%) = 1.02 × JDS (%) + 0.25%.^57^ T2D in subjects was defined as either a positive response to a questionnaire, prescription of a diabetes medication, or an HbA1c over 6.5%. Mitochondrial genetic variants were captured as described previously.^58^^,^^59^ Briefly, mitochondrial polymorphisms were determined with sequence-specific oligonucleotide probes (G&G Science, Fukushima, Japan) by use of suspension array technology (Luminex 100). The subjects without following data were excluded from the analyses: HbA1c (*n* = 6), MOTS-c genotype (m.1382A>C, *n* = 31), physical activity levels (*n* = 174), height (*n* = 1), and body fat percentage (*n* = 3). Consequently, 11,853 subjects were included in the final analyses (Tables S12 and S19–S22).

*J-MICC 2*^*nd*^*study*: Skeletal muscle mass and hand grip strength were assessed in the secondary survey in the Saga J-MICC study which was conducted five years after the first survey.^60^ This study consisted of 8,454 participants. Briefly, skeletal muscle mass was measured by using an S-BIS device (MLT-30, SK Medical, Electronics Co., Ltd., Shiga, Japan).^61^ The skeletal muscle mass index (kg/m^2^) was calculated by correcting the skeletal muscle mass by the square of the height. For hand grip strength measurement, a dynamometer (Grip-D, T.K.K. 5401, Takei Scientific Instruments, Niigata, Japan) was used to record the left- and right-hand grip strength values. The hand grip strength was recorded with the participant in a standing posture with the elbows extended, the hand grip strength values were measured once on each side, and the higher hand grip strength value (from either the right or left side) was used for the analysis. Among the 8,454 participants, those with insufficient data pertaining to the exposure and outcome variables were excluded; these included the lack of height (*n* = 29), body mass subtracted by clothe (*n* = 9), MOTS-c genotype (m.1382A>C, *n* = 17), hand grip strength (*n* = 35), and insufficient data on physical activity (*n* = 138). For skeletal muscle mass analysis, we further excluded the subjects without S-BIS measurement (*n* = 94). Consequently, 8,226 (men, 3,382; women, 4,844) and 8,132 (men, 3,353; women, 4,779) participants were included in the analyses for hand grip strength and skeletal muscle mass, respectively (Tables S12 and S21).

### Method details

#### Dot blot assay

The binding between MOTS-c and CK2 was examined by dot blot assay with MOTS-c (GenScript), CK2 complex (NEB), CK2α (GenScript), and CK2β (IBL). Each protein, 0–21.7 μg of MOTS-c, 0–1000 units of CK2 complex, 0–210 ng of CK2α, and/or 0–210 ng of CK2β, was dotted on nitrocellulose membrane (BioRad) and dried for 30 min at room temperature. Then, the membrane was blocked with SuperBlock (PBS) Blocking Buffer (Thermo Scientific) for 30 min at room temperature with gently shaking. Then, a protein solution with 5 μg/mL of each paired protein in SuperBlock (PBS) Blocking Buffer was flowed over the nitrocellulose membrane and incubated for 30 min at room temperature with gently shaking. Next, the membrane was washed three times for 5 min using 0.1% TBST, and then incubated with each primary antibody, MOTS-c (1:1000, YenZym, custom antibody), CK2α (1:1000, Cell Signaling Technology), or CK2β (1:1000, Invitrogen), in SuperBlock (PBS) Blocking Buffer for 30 min at room temperature with gently shaking. The membrane was washed three times for 5 min using 0.1% TBST, and then 1:10000 anti-rabbit secondary antibody (1:10000, Cell Signaling Technology) was added to the membrane tray and incubated for 30 min at room temperature with gently shaking, followed by three additional washing steps with 0.1% TBST. Clarity Western ECL substrate and Clarity Max Western ECL Substrate (Bio-Rad) were used for detecting specific dots. Membranes were imaged on a Bio-Rad ChemiDoc XRS + imager and Image Lab software (Bio-Rad).

#### Binding simulation

The binding between MOTS-c and CK2α was simulated by using AlphaFold2 and AlphaFold2-Multiper.^26^^,^^27^ The amino acid sequences of MOTS-c and CK2α were used as input amino acid sequences for ColabFold.^28^ ColabFold generated automatically generated the simulation of the binding between MOTS-c and CK2α, including detailed docking sites. For this simulation, default setting was used.

#### Surface plasmon resonance

The kinetics of MOTS-c WT/K14Q (GenScript) and CK2α (GenScript)/β (IBL) were assessed by using surface plasmon resonance (Biacore T200, Cytiva). MOTS-c or K14Q MOTS-c at 10 μg/mL concentration in immobilization buffer (10 mM sodium acetate, pH 5.0) was immobilized on Sensor Chip CM5 (Cytiva Life Sciences). The final densities of WT and K14Q MTOS-c in resonance unit were 278 RU and 193 RU, respectively. First, 70 nM of CK2α (GenScript) or CK2β (IBL) was flowed over to check the bingeing between MOTS-c and CK2α or CK2β. Then, since we detected the binding signal only with CK2α, CK2α was flowed (30 μL/min) over the sensor chip at 2.5 nM, 5.0 nM, 10 nM, and 20 nM in running buffer (0.01M HEPES, 0.15 M NaCl, 0.3 mM EDTA, and 0.05% tween). After each concentration of CK2α was assessed, CK2α was removed using 4 mM and 5 mM Sodium Hydroxide for MOTS-c and K14Q MOTS-c, respectively. Fitting results were assessed using chi-squared goodness of fit (Biacore T200 Evaluation Software, Cytiva Life Sciences).

#### Cell-free CK2 activity assay

In the cell-free condition, CK2 activity was assessed by using a Kinase-Glo Luminescent Kinase Assays (Promega) in a white clear bottom 96-well plate (Corning Incorporated, Corning, NY). In this cell-free kinase activity assay, we used a specific CK2 substrate peptide for kinase assay (MilliporeSigma). The reaction mixture contained the following: CK2 (1.5 ng/μL; NEB), 50 μM CK2 substrate peptide (MilliporeSigma), and CK2 buffer [40 mM Tris-Hcl (pH 7.5), 10 mM MgCl2, 0.5 mM dithiothreitol, and 150 mM NaCl]. 5 μL of desired concentration of MOTS-c were added to the 96 well plate, then, 20 μL of the reaction mixture containing kinase and substrate were added. The reaction was started by adding 25 μL ATP (final 3 μM). After incubation at 37°C for 1 h, 50 μL of Kinase-Glo Luminescent Kinase Assay reagent (Promega) was added, and luminescence was detected to determine the amount of the remaining ATP. MOTS-c does not affect luciferase activity directly. Kinase activity was determined by calculating percentage of utilized ATP by kinase [100% - percentage of the remaining ATP].

#### CK2 activity assay in cells and tissues

To assess CK2 activity in cells and tissues, we detected proteins by using western blot technique with a phospho-CK2 substrate antibody (Cell Signaling Technology) that recognizes endogenous proteins containing a pS/pTDXE motif, which is a CK2 phosphorylation consensus sequence. Thus, this antibody can comprehensively assess CK2 activity in cells and tissues. After the western blot imaging, we quantified all the detected bands in a well and used the total signal intensity adjusted by a loading control as a CK2 activity. The details about western blot protocol are in the following section.

#### Western blots

Tissue and cells were lysed with RIPA buffer containing the Halt protease and phosphatase inhibitor cocktail (Thermo Scientific); this mixture was then homogenized, incubated, and sonicated. Lysis supernatant was collected by centrifugation at 13,500 rpm for 15 min at 4°C. Protein content in the lysate was quantified by the Pierce BCA Protein Assay kit (Thermo Scientific). Predetermined amounts of proteins (30 μg) were separated on 8%–16% or 4%–20% SDS-PAGE gels and blotted onto PVDF membranes (Bio-Rad) by using the Trans-Blot Turbo Transfer System (Bio-Rad). Membranes were blocked with 5% bovine serum albumin or 5% non-fat dry milk (for GAPDH) Tris-buffered saline with 0.1% Tween 20 and incubated with diluted primary antibodies for phospho-CK2 substrate [(pS/pT)DXE] (1:1,000, Cell Signaling Technology), CK2α (1:1000, Cell Signaling Technology), CK2β (1:1000, Invitrogen), phospho-Akt Ser473 (1:1000, Cell Signaling Technology), Phospho-Akt Ser129 (1:1000, abcam), (1:1000, Cell Signaling Technology), total AKT (1:1000, Cell Signaling Technology), phospho-CDC37 Ser13 (1:1000, Cell Signaling Technology), total CDC37 (1:1000, Cell Signaling Technology), GAPDH (1:2000, Cell Signaling Technology), α-tubulin (1:1,000, abcam) at 4°C overnight. For the MOTS-c, 30 μg proteins were loaded into the 4–12% NuPAGE Bis-Tris protein gel, run with the MES SDS Running Buffer (Invitrogen), and transferred onto PVDF membranes by using the Trans-Blot Turbo Transfer System (Bio-Rad). Membranes were blocked with 5% bovine serum albumin Tris-buffered saline with 0.1% Tween 20 and incubated with diluted primary antibody against MOTS-c (1:1000, custom polyclonal antibody; YenZym) at 4°C overnight. This custom MOTS-c antibody (rabbit polyclonal, YenZym) was developed against human MOTS-c sequence (MRWQEMGYIFYPRKLR) and is a unique antibody in our group^12^ and differs from the one from the Lee lab.^6^ After several washes with Tris-buffered saline containing 0.1% Tween 20, the membranes were incubated at room temperature for 1 h with the appropriate HRP-conjugated secondary antibody (Cell Signaling Technology). Clarity Western ECL substrate and Clarity Max Western ECL Substrate (Bio-Rad) were used for detecting specific bands. Membranes were imaged on a Bio-Rad ChemiDoc XRS + imager and relative intensities of the bands were quantified using Image Lab software (Bio-Rad).

#### Coimmunoprecipitation assay

The gastrocnemius muscle, epididymal fat, and liver samples from control or MOTS-c administered (7.5 mg/kg) mice were used for coimmunoprecipitation assay. The tissues were collected 30 min after the administration. CK2α was immunoprecipitated from mouse skeletal muscle and fat tissues using CK2α polyclonal antibody (Bethyl Laboratories). Before immunoprecipitation, proteins were extracted from mouse gastrocnemius muscle, liver, and fat tissues using the RIPA buffer containing the Halt protease and phosphatase inhibitor cocktail (Thermo Scientific) (same protocol to the Western Blots). Then, 24 μg of CK2α (Bethyl Laboratories) or rabbit IgG (Cell Signaling Technology) antibody was conjugated to Dynabeads Protein G (Thermo Scientific) for 10 min rotating followed by one wash, as outlined in the manufacture protocol. After antibody conjugation, 2 mg extracted proteins from gastrocnemius muscle, epididymal fat, and liver were added to the antibody-conjugated beads for 10 min at room temperature rotating. Then, the beads were washed three times using the manufacture-provided wash buffer, and proteins were eluted for 5 min at 95°C using premixed sample buffers suitable for Invitrogen NuPAGE western blot. Supernatants were then loaded into the 4–12% NuPAGE Bis-Tris protein, gel run with the MOPS SDS Running Buffer (Invitrogen), and transferred onto PVDF membranes by using the Trans-Blot Turbo Transfer System (Bio-Rad). Then, the same protocol as the Western blots was used for detection. To avoid strong signals from light and heavy chains, we used an HRP-conjugated antibody for CK2α detection (Santa Cruz).

#### Interactome analysis

To analyze CK2-binding proteins with/without MOTS-c in muscle and fat, we collected mouse gastrocnemius muscle and fat 30 min after MOTS-c or sterilized water administration. Proteins were extracted from muscle and fat using the same protocols to the Western Blots, and coimmunoprecipitation was performed by using same protocols to the Coimmunoprecipitation assay with CK2α polyclonal antibody (Bethyl Laboratories) or rabbit IgG (Cell Signaling Technology). Immunoprecipitated samples were used for mass spectrometry analysis.

Samples were mixed with same volume of digestion buffer (8M Urea, 0.1M Tris-HCl pH 8.5), then each sample was reduced and alkylated via sequential 20-min incubations with 5 mM TCEP and 10 mM iodoacetamide at room temperature in the dark while being mixed at 1200 rpm in an Eppendorf thermomixer. 10 μL of carboxylate-modified magnetic beads (CMMB and also widely known as SP3^62^) was added to each sample. Ethanol was added to a concentration of 50% to induce protein binding to CMMB. CMMB were washed 3 times with 80% ethanol and then resuspended with 50 μL of 50 mM TEAB. The protein was digested overnight with 0.1 μg LysC (Promega) and 0.8 μg trypsin (Thermo Scientific) at 37°C. Following digestion, 1 mL of 100% acetonitrile was added to each to sample to increase the final acetonitrile concentration to over 95% to induce peptide binding to CMMB. CMMB were then washed 3 times with 100% acetonitrile and the peptide was eluted with 50 μL of 2% DMSO. Eluted peptide samples were dried by vacuum centrifugation and reconstituted in 5% formic acid before analysis by LC-MS/MS.

Peptide samples were separated on a 75 μM ID, 25cm C18 column packed with 1.9 μM C18 particles (Dr. Maisch GmbH) using a 140-min gradient of increasing acetonitrile concentration and injected into a Thermo Orbitrap-Fusion Lumos Tribrid mass spectrometer. MS/MS spectra were acquired using Data Dependent Acquisition (DDA) mode. MS/MS database searching was performed using MaxQuant^63^ (1.6.10.43) against the Mus musculus reference proteome from EMBL (UP000000589).

Statistical analysis of MaxQuant label-free quantitation data was performed with the artMS Bioconductor package which performs the relative quantification of protein abundance using the MSstats Bioconductor package^64^ (default parameters). The abundance of proteins missing from one condition but found in more than 2 biological replicates of the other condition for any given comparison were estimated by imputing intensity values from the lowest observed MS1-intensity across samples^65^ and *p*-values were randomly assigned to those between 0.05 and 0.01 for illustration purposes. To perform enrichment analyses, the proteins were filtered with an adjusted *p*-value <0.05 and fold change >2.0. The STRING database is used to conduct enrichment analyses and to visualize the protein-protein interaction network.^43^ For the enrichment analyses, the Reactome pathway database was utilized with false discovery rate (FDR) < 0.05.

#### *In vitro* and *in vivo* 2-deoxyglucose uptake (2-DG) assay

##### In vitro

To measure glucose uptake in fully differentiated human skeletal muscle cells, a 2-DG uptake measurement kit was used (Promega, Cat No. J1342). Briefly, 15 min after incubation in the cell culture media containing 1 μM of insulin (MilliporeSigma) and 10 μM of WT or K14Q MOTS-c (GenScript) at 37°C in 5% CO2, 2-DG was added to each well with a final concentration of 1 mM. Then, the cells were incubated for 30 min and used for the 2-DG uptake assay according to the manufacturer’s instructions (Promega).

##### In vivo

To measure glucose uptake into mouse skeletal muscle, 2-DG uptake measurement kit was used (Cosmo Bio). Gastrocnemius muscle samples were homogenized in 10 mM tris-HCL (pH. 8.1) and then incubated at 95°C for 15 min. Samples were centrifuged for 15 min at 17,800 g (4°C), and the supernatant was diluted in 10 mM tris-HCl.^66^ Diluted samples (20 mL) were used for further analysis according to the manufacturer’s instructions (Cosmo Bio Co.).

#### RNA extraction and RNA sequencing (RNA-seq) analysis

To understand the effect of the MOTS-c SNP on skeletal muscle gene expression levels, we collected skeletal muscle samples from Japanese individuals and performed RNA-seq analysis. Total RNA was extracted from muscle samples using TRIzol Reagent (Thermo Scientific). RNA concentration and purity were checked using a NanoDrop 8000 UV-Vis Spectrophotometer (Thermo Scientific). The RNA samples were sent to Novogene Co., LTD (Singapore). The RNA library was constructed by using a library preparation kit (NEBNext Ultra II Directional RNA Library Prep Kit for Illumina) and the prepped samples were sequenced on an Illumina Novaseq6000 by Novogene Co., LTD.

We also performed RNA-seq in WT or K14Q treated mouse skeletal muscles with immobilization to compare the effect of WT or K14Q MOTS-c treatment on gene expression levels in skeletal muscle. Total RNA was extracted from frozen gastrocnemius muscle by using TRIzol (Thermo Scientific), followed by the Quick-RNA Miniprep Kit (Zymo Research High quality RNA used for library preparation (mRNA-Seq Nu Quant). From there, prepped samples were sequenced on an Illumina NextSeq platform.

High quality FASTQ files were ensured using FastQC (v0.11.9) and mapped to the Human reference genome (GRCh38) or mouse reference genome (GRCm38) using STAR (v2.7.5c).^40^ Aligned sorted BAM files were inputted into R (v4.2.2) for counting. Count matrices were generated using the summarizeOverlaps function of the GenomicAlignements (v1.34.1) package.^41^ Differentially expressed genes (DEGs) between the genotypes or treatments were identified using DESeq2 package (v1.38.3)^42^ in R with a cut-off FDR of 0.05. Age and sex were used as covariates. Kyoto Encyclopedia of Genes and Genomes (KEGG) enrichment analysis of DEGs were performed to understand the biological functions by using Metascape.^44^ The significant enriched KEGG terms were identified with *p* < 0.05. The genotype of MOTS-c SNP, m.1382A>C, was visually determined by using the Integrative Genomics Viewer and we found five of 48 subjects had the C allele.

### Quantification and statistical analysis

Cell-free and mouse experiments: All data are expressed as mean ± SE. The Shapiro–Wilk test was used to assess the normality of the parameters and significant differences were determined by the unpaired t-tests or Wilcoxon rank-sum test. WASEDA’S Health Study: The prevalence of low skeletal muscle mass assessed by ASMI was tested using the chi-square test. The association of the MOTS-c K14Q polymorphism with continuous variables were assessed by two-way analysis of covariance (ANCOVA) adjusted for age and sex. We tested MOTS-c K14Q × sex or MVPA group interaction to assess the effect modification by sex and MVPA group. The association of the MOTS-c K14Q polymorphism and MVPA group with the prevalence of low muscle mass was assessed by logistic regression analysis adjusted for age and sex, and the multivariate-adjusted odds ratios (ORs) and 95% confidence intervals (CIs) were calculated using the A allele as reference. J-MICC: The prevalence of T2D, categorized based on the MOTS-c SNP, was compared using the chi-square test. The risk of T2D was examined by using multivariable regression analysis and MOTS-c SNP, age, sex, and/or MVPA were used as covariates. The skeletal muscle mass and grip strength, categorized based on the MOTS-c SNP, were compared by ANCOVA adjusted for BMI and MVPA. Statistical significance was set at *p* < 0.05. Statistical analyses were performed using R programming environment (v4.0.3) using R Studio (v1.4.1103), JMP Pro (version 16.2.0; SAS Institute Inc.), and SPSS Statistics (version 29.0; SPSS, Inc.).

## Supplemental information

Document S1. Figures S1–S13, Tables S9, S10, S12, S13, and S15–S22

Table S1. List of proteins bind to CK2α in skeletal muscle in control group, related to Figure 4D

Table S2. List of proteins bind to CK2α in skeletal muscle in MOTS-c group, related to Figure 4D

Table S3. List of proteins bind to CK2α in fat in control group, related to Figure 4F

Table S4. List of proteins bind to CK2α in fat in MOTS-c group, related to Figure 4F

Table S5. List of enriched Reactome pathways in the distilled water-administered mouse skeletal muscle, related to Figure 4E

Table S6. List of enriched Reactome pathways in the MOTS-c-administered mouse skeletal muscle, related to Figure 4E

Table S7. List of enriched Reactome pathways in the distilled water-administered mouse, fat related to Figure 4G

Table S8. List of enriched Reactome pathways in the MOTS-c-administered mouse fat, related to Figure 4G

Table S11. Differentially regulated genes between WT and K14Q-treated gastrocnemius muscles in immobilized mice, related to Figure 5F

Table S14. Differentially regulated genes in vastus lateralis muscles from Japanese carriers of the m.1382A>C polymorphism: Comparison of A (WT) or C (K14Q) allele carriers, related to Figure 6A-B
