# Supplementary material for: MOTS-c modulates skeletal muscle function by directly binding and activating CK2
Source: iScience. 2024 Oct 19;27(11):111212. doi: 10.1016/j.isci.2024.111212 (PMC11570452; doi:10.1016/j.isci.2024.111212)
Supplement: Document S1. Figures S1–S13, Tables S9, S10, S12, S13, and S15–S22 [file mmc1.pdf]

iScience, Volume *27*

Supplemental information

MOTS-c modulates skeletal muscle function by directly binding and activating CK2

Hiroshi Kumagai, Su-Jeong Kim, Brendan Miller, Hirofumi Zempo, Kumpei Tanisawa, Toshiharu Natsume, Shin Hyung Lee, Junxiang Wan, Naphada Leelaprachakul, Michi Emma Kumagai, Ricardo Ramirez II, Hemal H. Mehta, Kevin Cao, Tae Jung Oh, James A. Wohlschlegel, Jihui Sha, Yuichiro Nishida, Noriyuki Fuku, Shohei Dobashi, Eri Miyamoto-Mikami, Mizuki Takaragawa, Mizuho Fuku, Toshinori Yoshihara, Hisashi Naito, Ryoko Kawakami, Suguru Torii, Taishi Midorikawa, Koichiro Oka, Megumi Hara, Chiharu Iwasaka, Yosuke Yamada, Yasuki Higaki, Keitaro Tanaka, Kelvin Yen, and Pinchas Cohen

MOTS-c modulates skeletal muscle function by directly binding and activating CK2

Hiroshi Kumagai[1)], Su-Jeong Kim[1)], Brendan Miller[1)], Hirofumi Zempo[2)], Kumpei Tanisawa[3)], Toshiharu Natsume[4)], Shin Hyung Lee[1)], Junxiang Wan[1)], Naphada Leelaprachakul[1)], Michi Emma Kumagai[1,5)], Ricardo Ramirez II[1)], Hemal H. Mehta[1)], Kevin Cao[1)], Tae Jung Oh[1,6)], James A Wohlschlegel[7)], Jihui Sha[7)], Yuichiro Nishida[8)], Noriyuki Fuku[9)], Shohei Dobashi[10)], Eri Miyamoto-Mikami[9)], Mizuki Takaragawa[9)], Mizuho Fuku[9,11)], Toshinori Yoshihara[9)], Hisashi Naito[9)], Ryoko Kawakami[12)], Suguru Torii[3)], Taishi Midorikawa[13)], Koichiro Oka[3)], Megumi Hara[8)], Chiharu Iwasaka[14)], Yosuke Yamada[14)], Yasuki Higaki[15)], Keitaro Tanaka[8)], Kelvin Yen[1)], Pinchas Cohen[1)*]

1) The Leonard Davis School of Gerontology, University of Southern California, California, USA
2) Department of Administrative Nutrition, Faculty of Health and Nutrition, Tokyo Seiei College, Tokyo, Japan
3) Faculty of Sport Sciences, Waseda University, Saitama, Japan
4) Faculty of Medicine, Tokai University, Kanagawa, Japan
5) Department of Psychiatry, David Geffen School of Medicine, University of California, Los Angeles, California, USA
6) Department of Internal Medicine, Seoul National University College of Medicine, Seoul, Korea
7) Department of Biological Chemistry, University of California, Los Angeles, California, USA.
8) Department of Preventive Medicine, Faculty of Medicine, Saga University, Saga, Japan
9) Graduate School of Health and Sports Science, Juntendo University, Chiba, Japan.
10) Institute of Health and Sport Sciences, University of Tsukuba, Ibaraki, Japan
11) Tsudanuma Central General Hospital, Chiba, Japan
12) Physical Fitness Research Institute, Meiji Yasuda Life Foundation of Health and Welfare, Tokyo, Japan
13) College of Health and Welfare, J.F. Oberlin University, Tokyo, Japan
14) Department of Physical Activity Research, National Institutes of Biomedical Innovation, Health and Nutrition, Osaka, Japan
15) Laboratory of Exercise Physiology, Faculty of Sports and Health Science, Fukuoka University, Fukuoka, Japan

*Corresponding author. Email: hassy@usc.edu

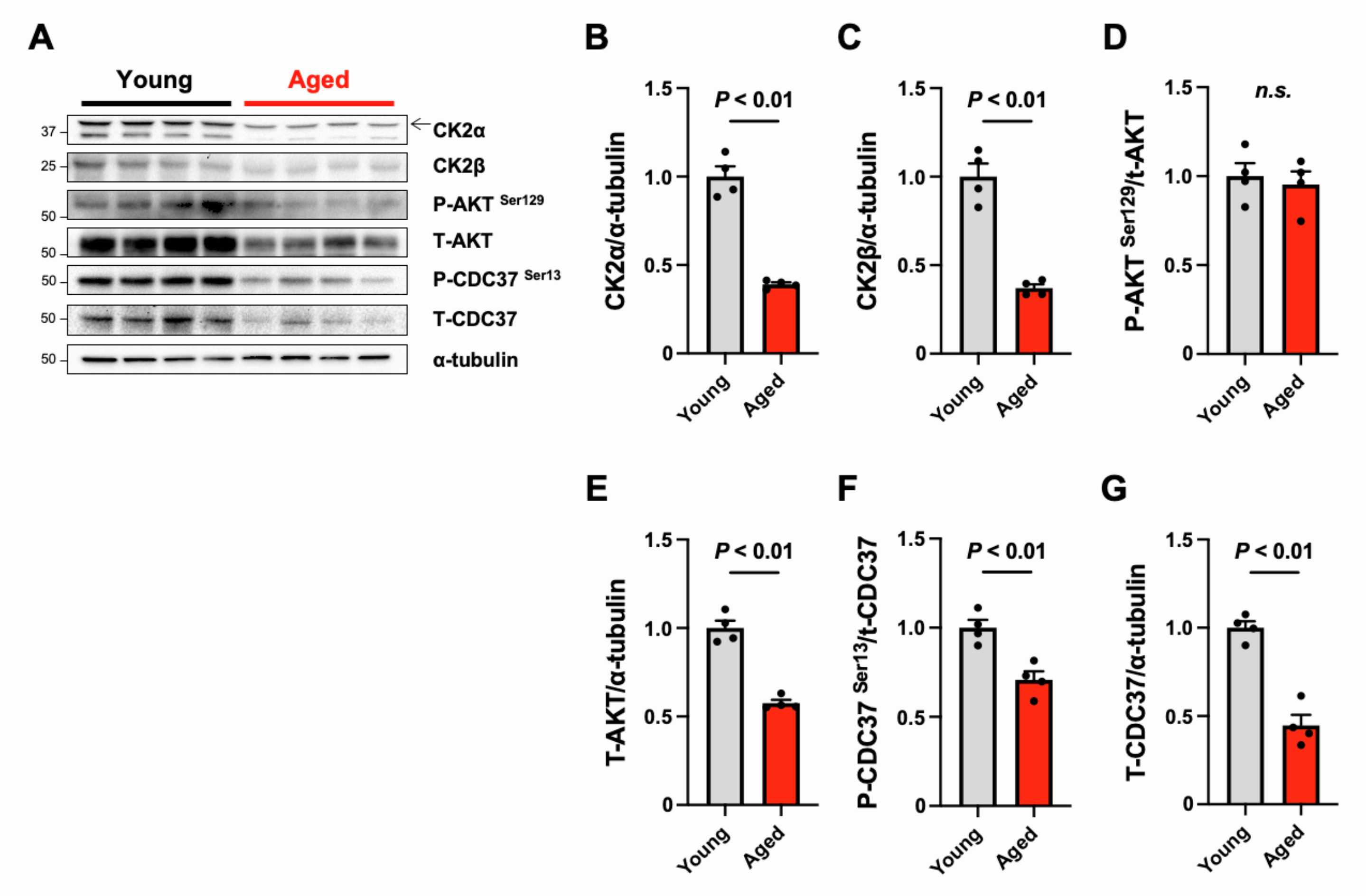

Supplemental-Figure 1. Expression levels of CK2α, CK2β, and direct substrates of CK2 in young and aged mice.

(A): Western blot images of the analyzed proteins in young (2 months) and aged (22 months) mice (n = 4 per group). **(B-G)**: Quantification of CK2α/α-tubulin **(B)**, CK2β/α-tubulin **(C)**, pAKT Ser129/total AKT **(D)**, total AKT/α-tubulin **(E)**, pCDC37 Ser13/total CDC37 **(F)**, and total CDC37/α-tubulin fat **(G)**.

Data are represented as mean +/- SEM.

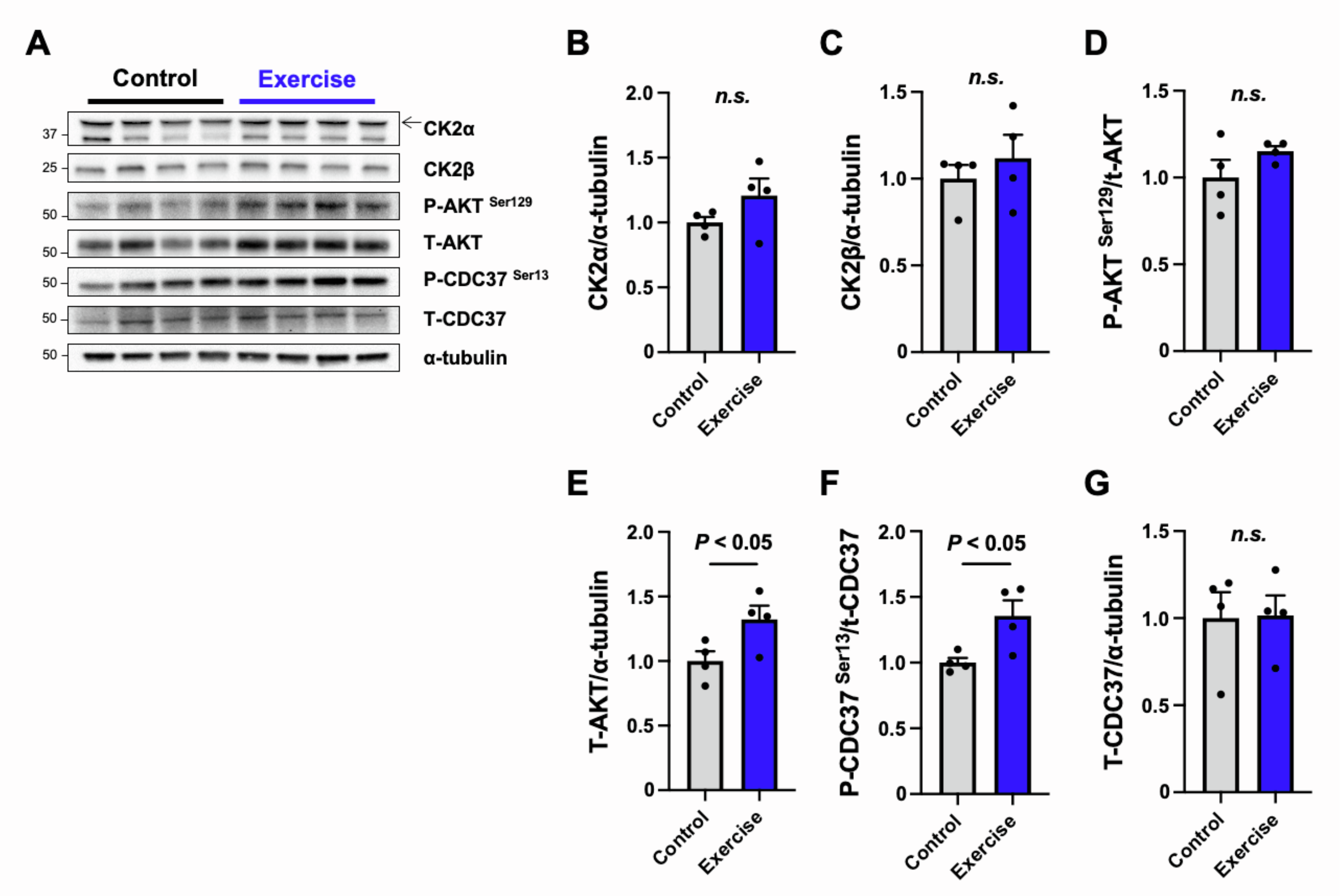

Supplemental-Figure 2. Expression levels of CK2α, CK2β, and direct substrates of CK2 in sedentary and exercised young mice.

(A): Western blot images of the analyzed proteins in sedentary and exercise trained mice (n = 4 per group). Mice in exercise training group were housed in cages with running wheels for 4 weeks. **(B-G)**: Quantification of CK2α/α-tubulin **(B)**, CK2β/α-tubulin **(C)**, pAKT Ser129/total AKT **(D)**, total AKT/α-tubulin **(E)**, pCDC37 Ser13/total CDC37 **(F)**, and total CDC37/α-tubulin fat **(G)**.

Data are represented as mean +/- SEM.

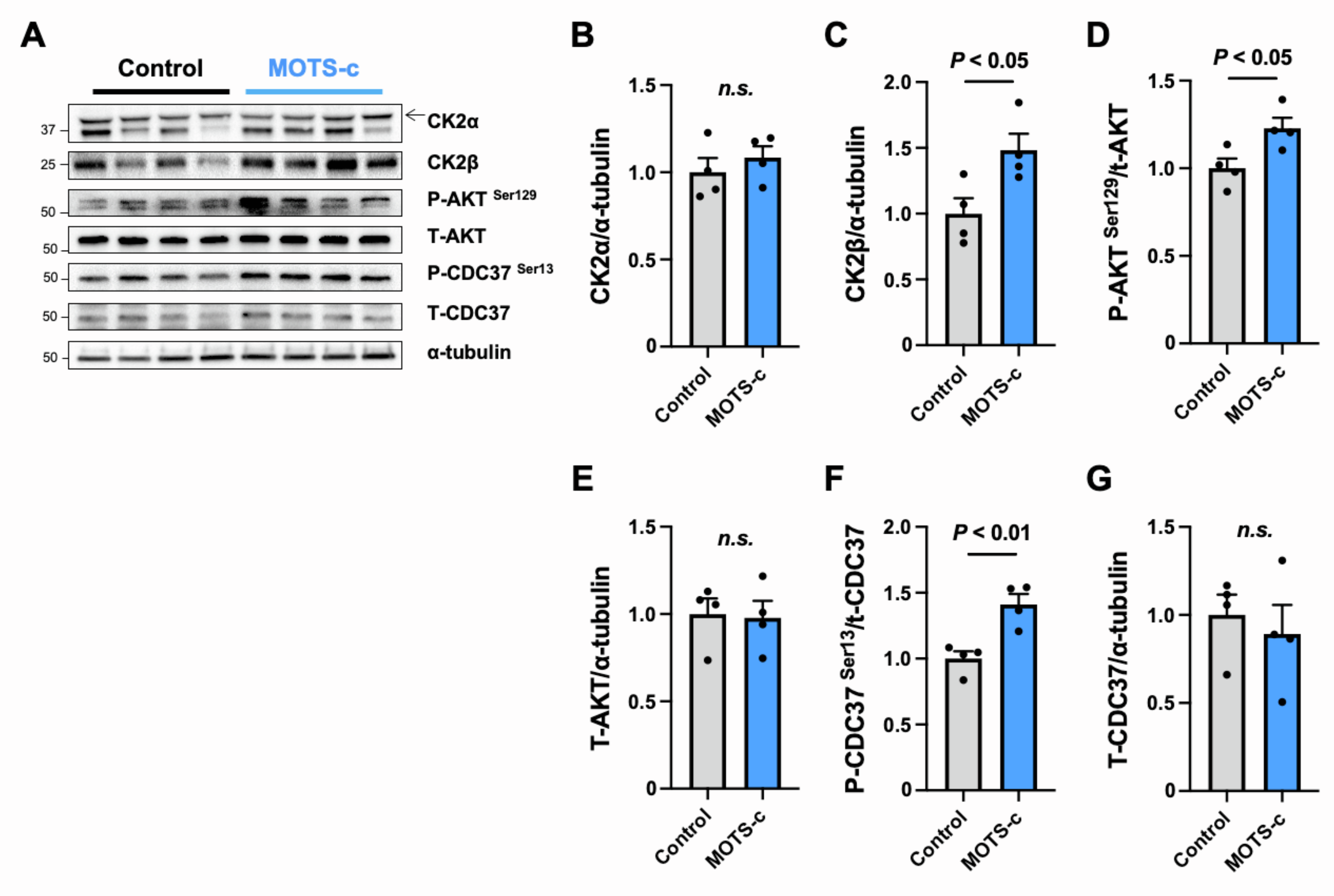

Supplemental-Figure 3. Expression levels of CK2α, CK2β, and direct substrates of CK2 in control and MOTS-c treated high-fat diet-fed (HFD) mice.

(A): Western blot images of the analyzed proteins in control and MOTS-c treated HFD-fed mice (n = 4 per group). MOTS-c was administered by IP injection twice a day for 8 weeks (5 mg/kg/day). **(B-G)**: Quantification of CK2α/α-tubulin **(B)**, CK2β/α-tubulin **(C)**, pAKT Ser129/total AKT **(D)**, total AKT/α-tubulin **(E)**, pCDC37 Ser13/total CDC37 **(F)**, and total CDC37/α-tubulin fat **(G)**.

Data are represented as mean +/- SEM.

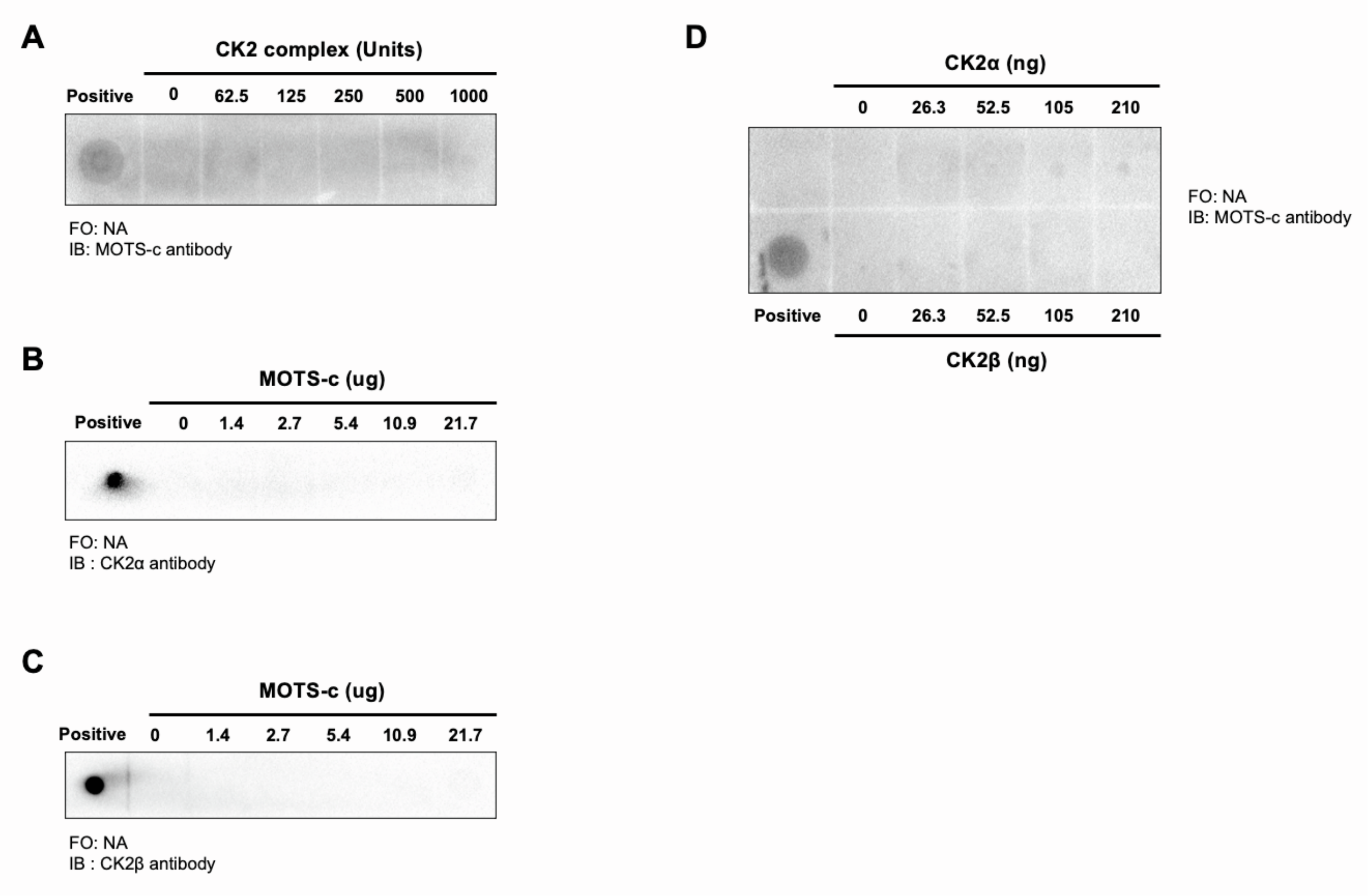

Supplemental-Figure 4. Dot blot images for assay controls.
(A-C): Dot blot assays with **(A)** CK2 complex (contains both CK2α and CK2β subunits) immobilized, flowed over the membrane without MOTS-c, and detected by MOTS-c antibody, **(B-C):** MOTS-c immobilized, flowed over the membrane without CK2 complex, and detected by CK2α **(B)** and CK2β **(C)** antibodies. **(D):** CK2α or CK2β immobilized, flowed over the membrane without MOTS-c, and detected by MOTS-c antibody. FO: flow over, IB: immunoblotting.
Positive controls: MOTS-c (1085 ng), CK2α (420 ng), and CK2β (200 ng).

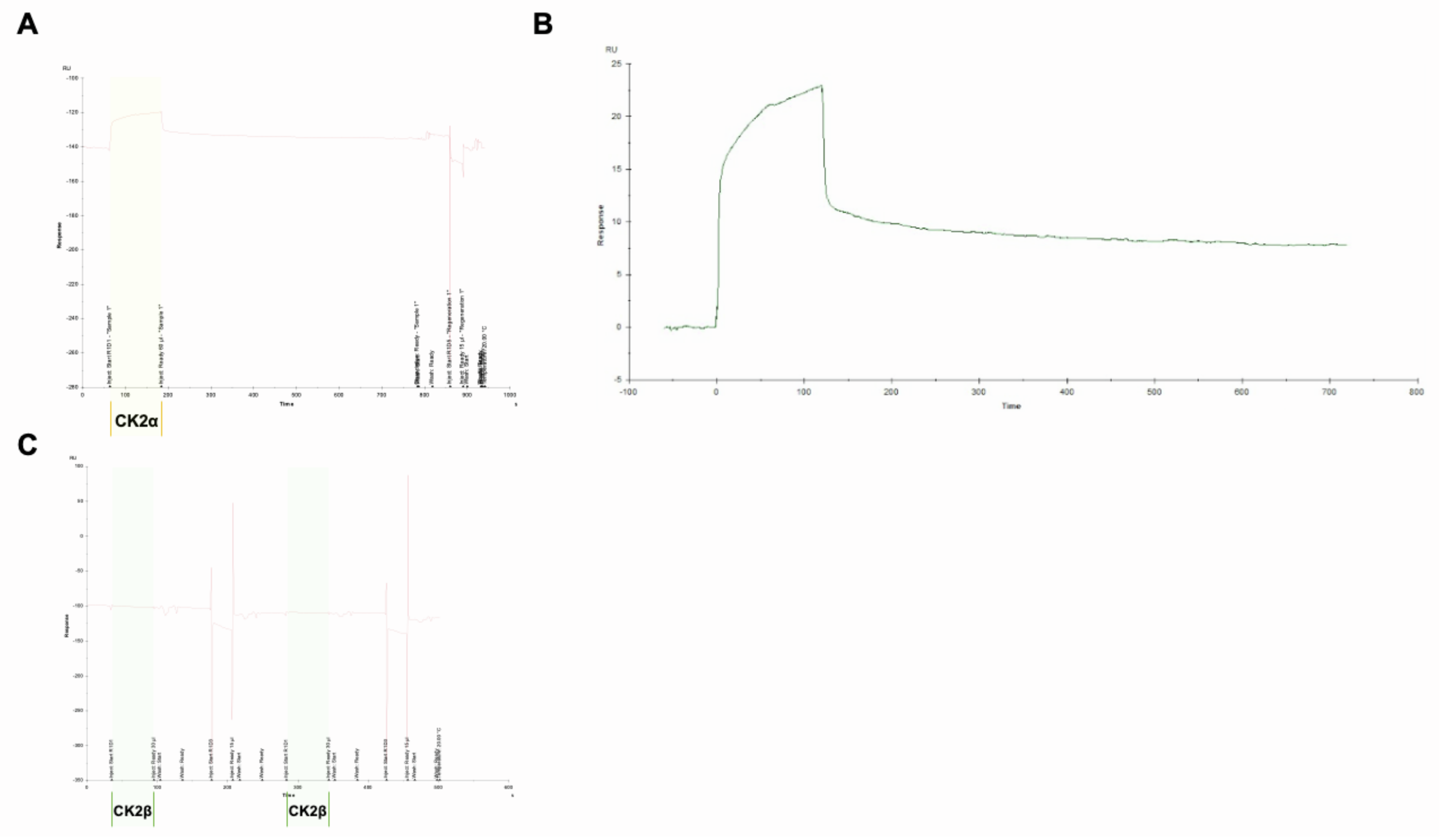

Supplemental-Figure 5. Surface plasmon resonance assay of MOTS-c and CK2 subunits

(A): Raw signal data of the surface plasmon resonance assay of MOTS-c and CK2α subunit. Running buffer flowed first, then CK2α (70 nM) flowed during the yellow highlighted period. **(B)**: Binding signal between MOTS-c and CK2α subtracted by the negative control signal. (C): Raw signal data of the surface plasmon resonance assay of MOTS-c and CK2β subunit. Running buffer flowed first, then CK2β (70 nM) flowed during the green highlighted period. CK2β flowed twice to confirm no binding.

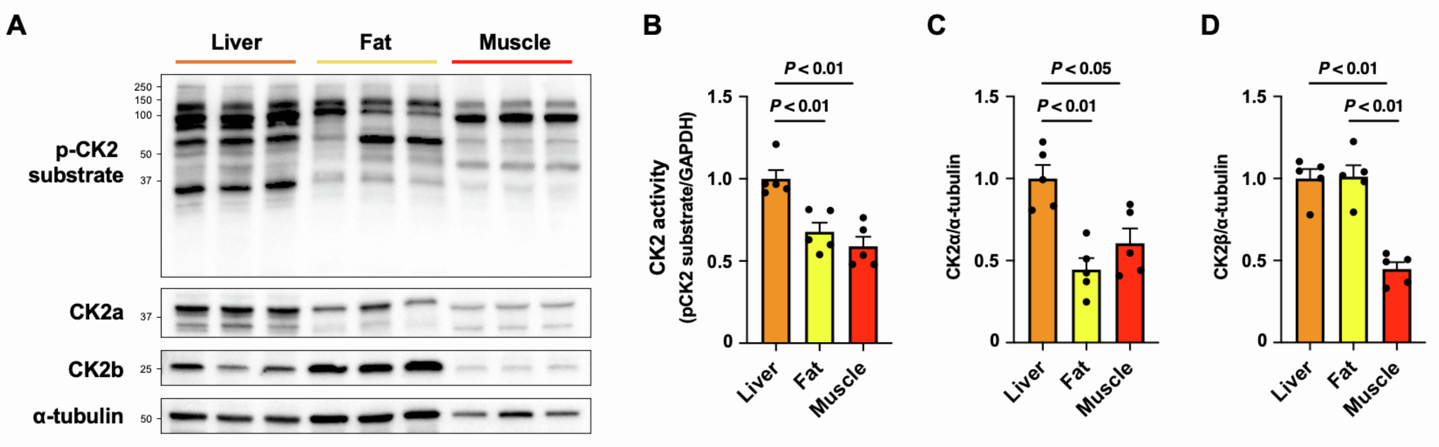

Supplemental-Figure 6. Comparison of CK2 activity and expression levels of CK2 subunits in liver, epididymal fat, and gastrocnemius muscle.

(A): Expression levels of phosphor-CK2 substrate, CK2α, and CK2β in liver, epididymal fat, and gastrocnemius muscle in young mice (n = 5). **(B-D)**: Quantifications of CK2 activity assessed by detecting phosphor-CK2 substrate, CK2α, and CK2β in liver, epididymal fat, and gastrocnemius muscle.

Data are represented as mean +/- SEM.

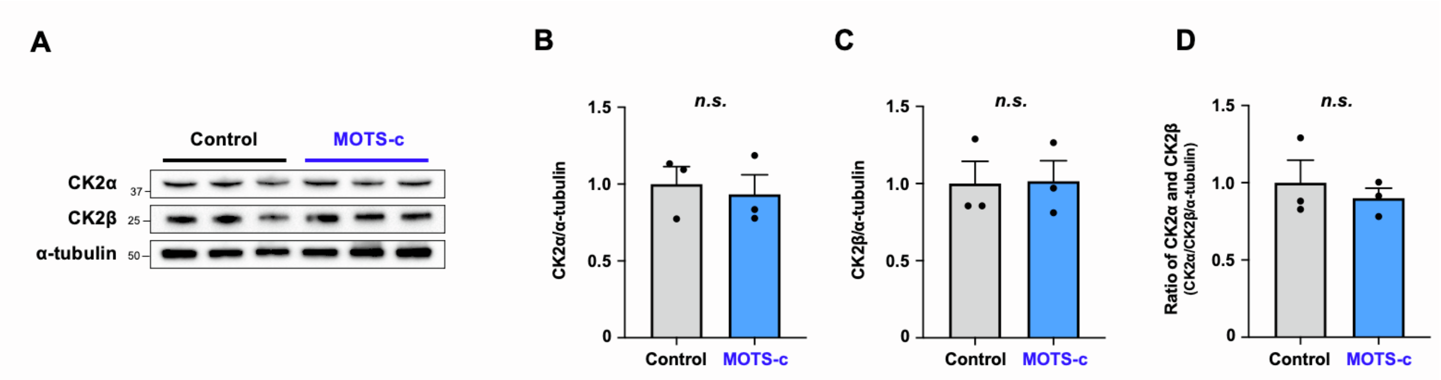

Supplemental-Figure 7. Effect of a single MOTS-c administration on CK2α and CK2β expression levels in gastrocnemius muscle.

(A): Western blot images of CK2α and CK2β in control and MOTS-c administered young mice (n = 3 per group). MOTS-c was administered by IP injection (7.5 mg/kg) 30 minutes before tissue collection. **(B-D)**: Quantifications of CK2α and CK2β in gastrocnemius muscle.

Data are represented as mean +/- SEM.

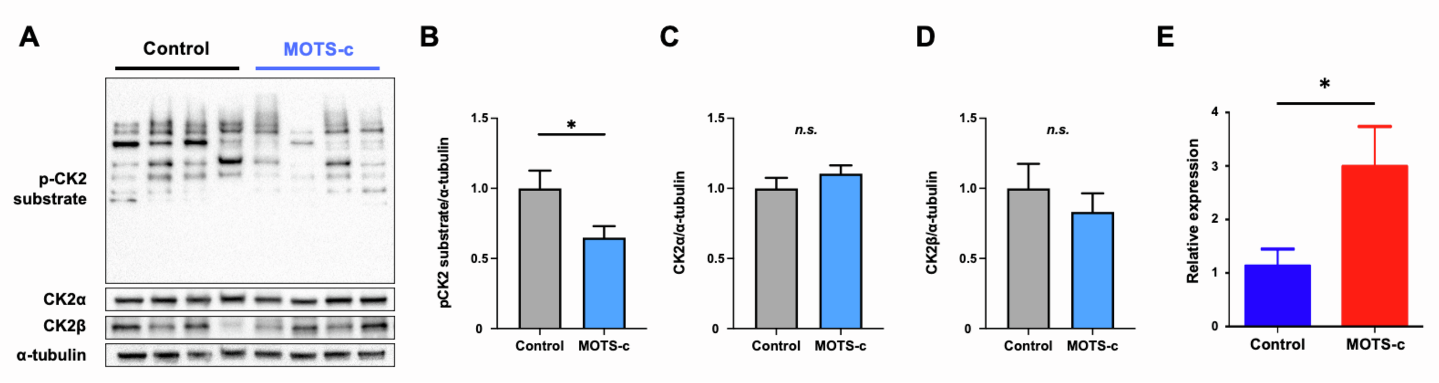

Supplemental-Figure 8. Effect of 8 weeks of MOTS-c treatment on CK2 in epididymal fat
(A-B): Western blot images of phospho-CK2 substrate as an index of CK2 activity, CK2α, and CK2β expression levels in epididymal fat. **(B-D)**: Quantification of phospho-CK2 substrate as an index of CK2 activity, CK2α, and CK2β expression levels in epididymal fat. **(E)**: Expression levels of UCP1 mRNA in epididymal fat.
Data are represented as mean +/- SEM.
*: $P < 0.05$.

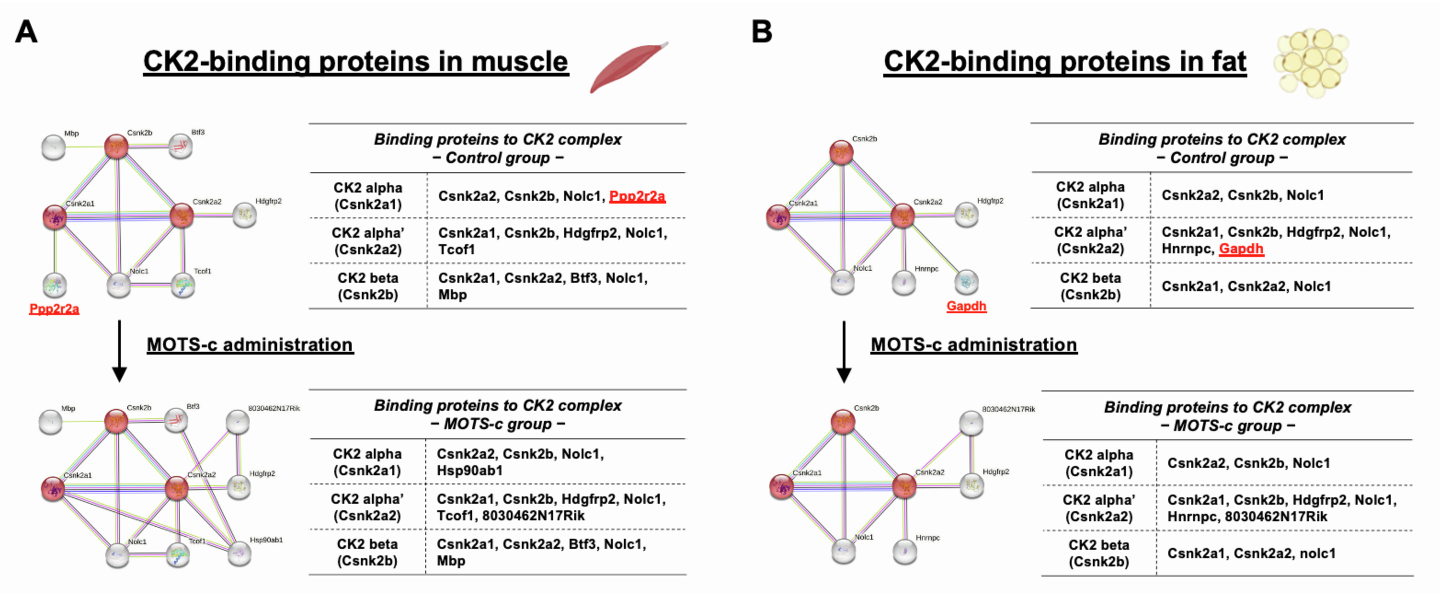

Supplemental-Figure 9. Direct interacting proteins with the MOTS-c/CK2 complex

(A-B): Direct interacting proteins with the MOTS-c/CK2 complex in gastrocnemius muscle **(A)** and epididymal fat **(B)** in control and MOTS-c administered mice. MOTS-c was administered by IP injection (7.5 mg/kg) 30 minutes before the tissue collection. The direct interacting proteins were analyzed by using the STRING database.

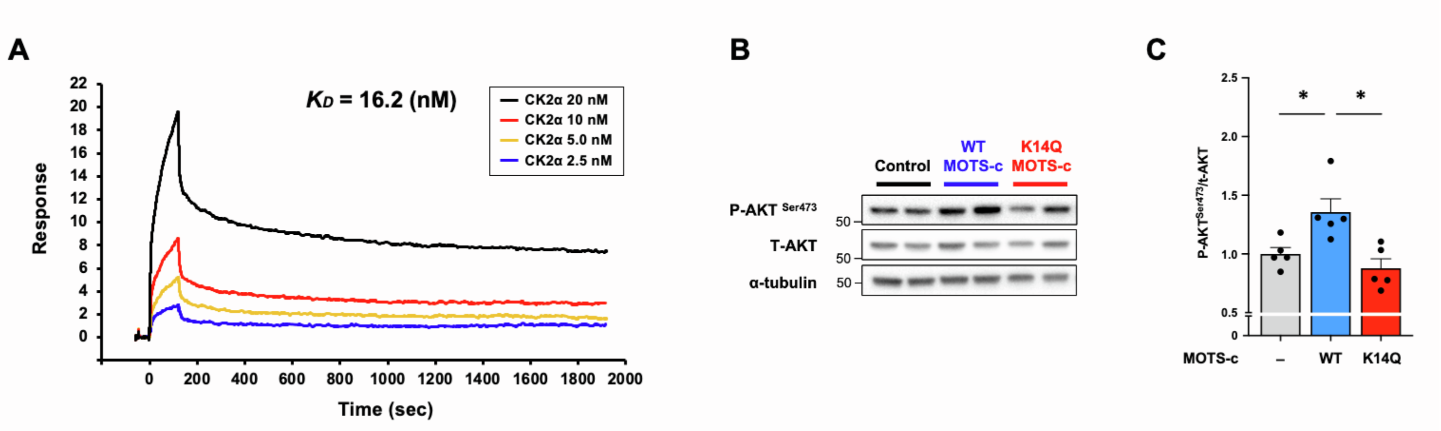

Supplemental-Figure 10. K14Q MOTS-c is a bio-inactive form of MOTS-c

(A): Surface plasmon resonance (Biacore assay) of K14Q MOTS-c (10 μg/ml) and CK2α (2.5 nM, 5.0 nM, 10 nM, and 20 nM). K14Q MOTS-c was immobilized on the sensor chip and CK2α flowed over the sensor chip. K_D: dissociation constant. **(B-C)**: Western blot result of phosphor-AKT Ser473 in WT MOTS-c and K14Q MOTS-c administered (2.5 mg/kg) mouse gastrocnemius muscles. MOTS-c was administered by IP injection 30 minutes before the tissue collection.

Data are represented as mean +/- SEM.

*: $P < 0.05$.

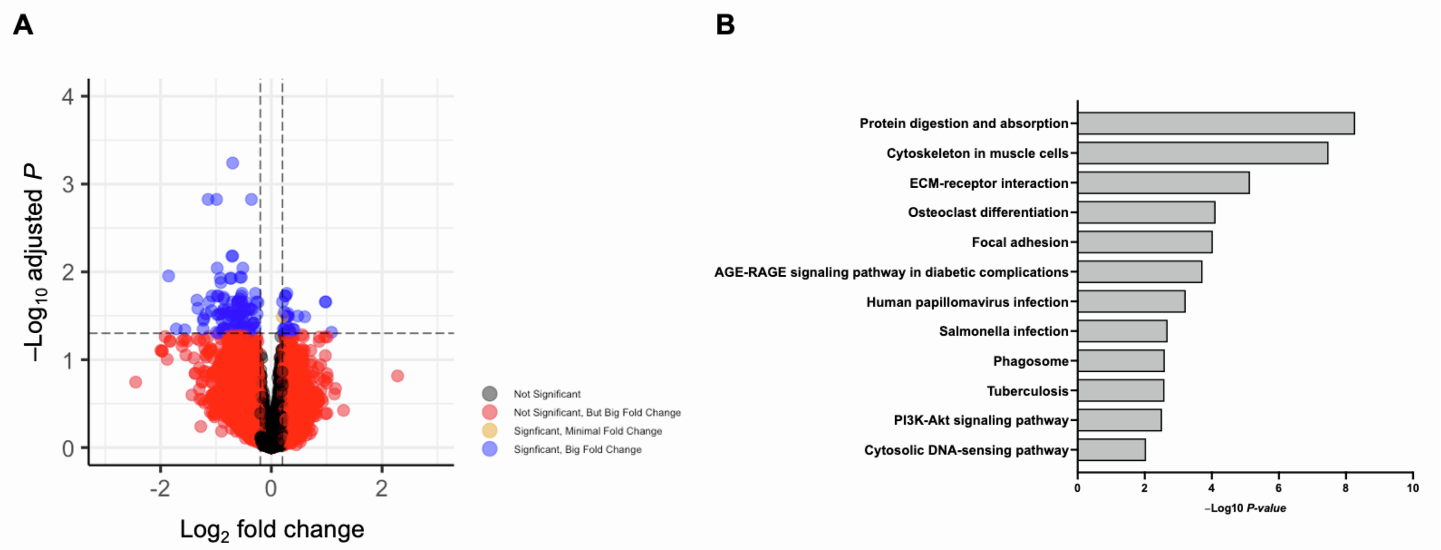

Supplemental-Figure 11. Skeletal muscle RNA sequencing in gastrocnemius muscles from WT or K14Q MOTS-c treated immobilized mice

(A): Differentially expressed genes between WT and K14Q MOTS-c treated mouse gastrocnemius muscles. Mice were immobilized for 8 days with WT or K14Q MOTS-c treatment. WT or K14Q MOTS-c was administered by IP injection twice a day (15 mg/kg/day). **(B)**: Significant enriched KEGG pathways between WT and K14Q MOTS-c treated mouse gastrocnemius muscles.

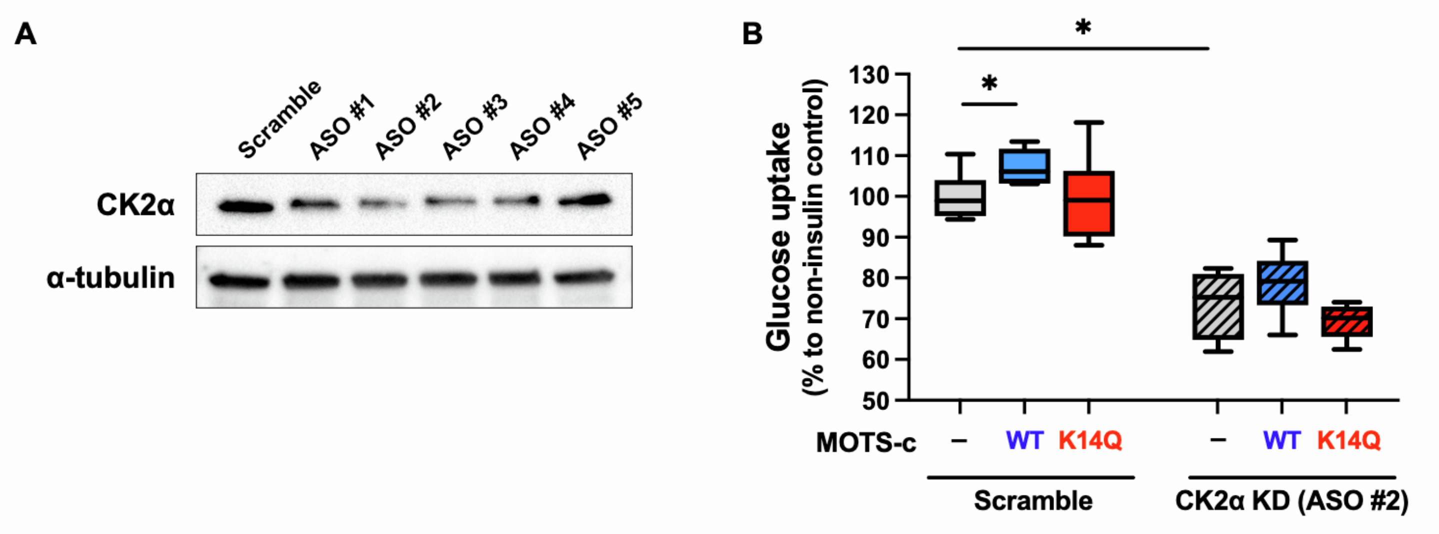

Supplemental-Figure 12. MOTS-c induced glucose uptake in differentiated human skeletal muscle cells with/without CK2 α knockdown (KD).

(A): Western blot image of CK2 α expression by in 5 different AUM*silence* ASO targeting CK2α and scramble control. Differentiated human skeletal muscle cells were incubated with each ASO (5 µM) for 48h for CK2α KD.

(B): 2-deoxyglucose (2-DG) uptake with/without CK2α KD in differentiated human skeletal muscle cells. The differentiated human skeletal muscle cells were incubated with scramble or ASO #2 (5 µM) for 24h to knockdown CK2α. Then, the cells were treated with insulin (1 µM), and WT or K14Q MOTS-c (10 µM) for 15 minutes, followed by 2DG uptake assay.

ASO: antisense oligonucleotide.

Data are represented as median and min to max.

*: $P < 0.05$.

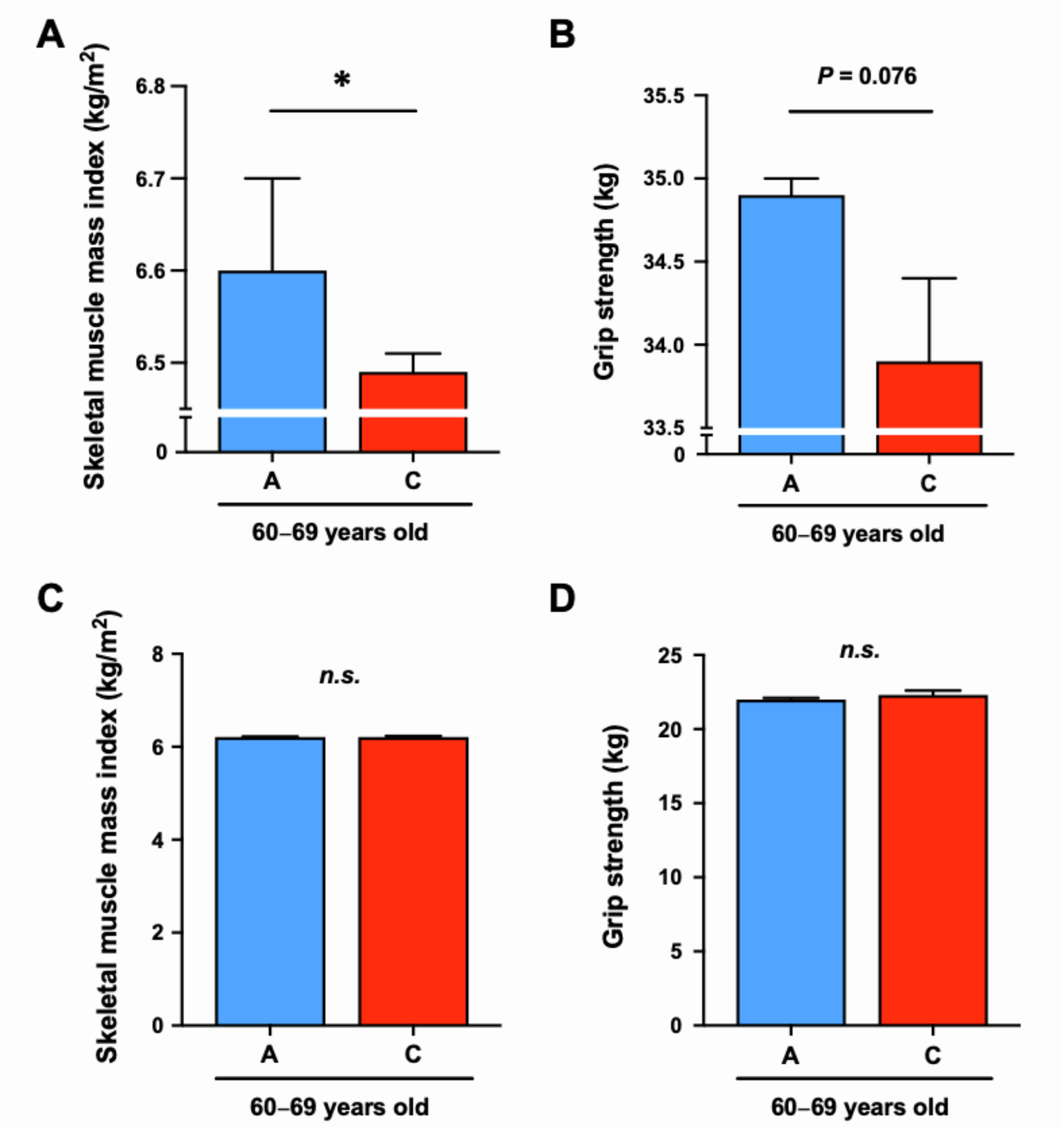

Supplemental-Figure 13. The bio-inactive K14Q MOTS-c decreases skeletal muscle mass index and muscle strength in aged population

(A-B): Skeletal muscle mass index **(A and C)** and grip strength **(B and D)** between the A allele and C allele carriers of m.1382A>C polymorphism in the aged males **(A-B)** and females **(C-D)**. The studied male subjects were from J-MICC 2nd study. The sample size for skeletal muscle mass index and grip strength were 8,132 and 8226, respectively.

Data are represented as mean +/- SEM.

*: $P < 0.05$.

Supplemental-Table 9. List of differentially expressed proteins bind to CK2α between distilled water- and MOTS-c-administered mouse skeletal muscle

Protein ID	Gene symbol	Protein Name	iLog2FC	iPvalue
P21107	Tpm3	tropomyosin 3, gamma	7.192069042	0.0174315
P28798	P28798	NA	4.540608767	0.0325498
P47754	Capza2	capping protein (actin filament) muscle Z-line, alpha 2	4.509815656	0.0318659
P58774	Tpm2	tropomyosin 2, beta	4.141239228	0.0250031
Q9D0R8	Lsm12	LSM12 homolog	4.058109575	0.0261857
Q8K4P0	Wdr33	WD repeat domain 33	3.702798176	0.0345665
O55126	O55126	NA	3.346322717	0.0113196
P97443	Smyd1	SET and MYND domain containing 1	3.29055197	0.0285672
P47757	Capzb	capping protein (actin filament) muscle Z-line, beta	3.244881991	0.0148672
A0A0B4J1K5	A0A0B4J1K5	NA	3.154180022	0.0351659
P09542	Myl3	myosin, light polypeptide 3	3.046810962	0.0160006
Q01149	Col1a2	collagen, type I, alpha 2	3.038274028	0.0290042
P27573	Mpz	myelin protein zero	2.773730354	0.0140203
P83741	Wnk1	WNK lysine deficient protein kinase 1	2.750540391	0.0327225
P20801	Tnnc2	troponin C2, fast	2.673346188	0.0300756
O08638	O08638	NA	2.66157645	0.0177936
E9PWQ3	E9PWQ3	NA	2.613920042	0.0474524
Q70IV5	Synm	synemin, intermediate filament protein	2.560987857	0.0480641
Q9CQB2	Mcrip2	MAPK regulated corepressor interacting protein 2	2.456683342	0.0278157
Q91WK2	Eif3h	eukaryotic translation initiation factor 3, subunit H	2.380124869	0.0177151
Q05920	Q05920	NA	2.310135725	0.0334907

Q9D1G3	Hhatl	hedgehog acyltransferase-like	2.261435557	0.0300571
Q5FW52	Mlip	muscular LMNA-interacting protein	2.112713449	0.0288336
Q8JZQ9	Eif3b	eukaryotic translation initiation factor 3, subunit B	2.079465927	0.0279597
Q8VDD5	Myh9	myosin, heavy polypeptide 9, non-muscle	2.056541792	0.0489364
Q02788	Col6a2	collagen, type VI, alpha 2	2.036779538	0.0270092
A0A075B5T9	A0A075B5T9	NA	1.742387835	0.0323141
Q05793	Hspg2	perlecan (heparan sulfate proteoglycan 2)	1.711665976	0.0190108
P23116	Eif3a	eukaryotic translation initiation factor 3, subunit A	1.634096354	0.0324915
P07356	Anxa2	annexin A2	1.594925998	0.0133197
P11531	Dmd	dystrophin, muscular dystrophy	1.09950916	0.0189157
Q9CZ13	Uqcrc1	ubiquinol-cytochrome c reductase core protein 1	-2.451408398	0.0486798

Comparison of skeletal muscle samples with CK2α immunoprecipitations between distilled water- and MOTS-c administered mice (iPvalue < 0.05).

Supplemental-Table 10. List of differentially expressed proteins bind to CK2α between distilled water- and MOTS-c-administered mouse fat

Protein ID	Gene symbol	Protein Name	iLog2FC	iPvalue
Q9D2G5	Synj2	synaptojanin 2	8.47421072	0.02386
Q9D824	Fip1l1	FIP1 like 1 (S. cerevisiae)	7.33678479	0.0112188
P98086	C1qa	complement component 1, q subcomponent, alpha polypeptide	6.23635549	0.0127687
Q60803	Traf3	TNF receptor-associated factor 3	5.02025413	0.0285897
Q8CF66	Lamtor4	late endosomal/lysosomal adaptor, MAPK and MTOR activator 4	5.00676528	0.0375359
P04945	P04945	NA	4.24843097	0.0441293
P41105	Rpl28	ribosomal protein L28	4.23120773	0.0424605
Q569Z6	Thrap3	thyroid hormone receptor associated protein 3	4.16011256	0.0391485
A0A075B5T3	A0A075B5T3	NA	4.15633863	0.0293884
A0A075B5X3	A0A075B5X3	NA	3.55559168	0.0225436
P56395	Cyb5a	cytochrome b5 type A (microsomal)	3.40703389	0.0145055
P11031	Sub1	SUB1 homolog, transcriptional regulator	3.40650789	0.0461463
A0A075B5M2	A0A075B5M2	NA	3.36905992	0.023844
Q8BH50	8030462N17Rik	RIKEN cDNA 8030462N17 gene	3.28230822	0.0115527
Q60870	Q60870	NA	2.95772798	0.0364198
A0A0A6YXA5	A0A0A6YXA5	NA	2.92729083	0.0291781
Q8QZT1	Acat1	acetyl-Coenzyme A acetyltransferase 1	2.47663512	0.0271226
Q61753	Phgdh	3-phosphoglycerate dehydrogenase	2.34621112	0.0315172
P62830	Rpl23	ribosomal protein L23	2.33112162	0.0328309
P11499	Hsp90ab1	heat shock protein 90 alpha (cytosolic), class B member 1	2.32483412	0.0363328
P61358	Rpl27	ribosomal protein L27	2.29098077	0.0256708
Q60931	Vdac3	voltage-dependent anion channel 3	2.26888962	0.0493133
P59708	Sf3b6	splicing factor 3B, subunit 6	2.20544218	0.012859
Q8VIJ6	Sfpq	splicing factor proline/glutamine rich (polypyrimidine tract binding protein associated)	2.14556419	0.0127245
Q8K310	Matr3	matrin 3	2.01271197	0.0135782
A0A075B5Y3	A0A075B5Y3	NA	1.94021358	0.0141639
P68254	Ywhaq	tyrosine 3-monooxygenase/tryptophan 5-monooxygenase activation protein theta	1.84077689	0.0388817
A0A075B5Y4	A0A075B5Y4	NA	1.80096261	0.0110165
Q02257	Jup	junction plakoglobin	1.61024499	0.0227959
P17897	Lyz1	lysozyme 1	1.30747545	0.0360416
Q60854	Serpinb6a	serine (or cysteine) peptidase inhibitor, clade B, member 6a	0.99888001	0.0344708

O08529	Capn2	calpain 2	-1.227369	0.0372956
P17156	Hspa2	heat shock protein 2	-1.2601388	0.0334563
Q05421	Cyp2e1	cytochrome P450, family 2, subfamily e, polypeptide 1	-1.7277712	0.0138868
Q99L88	Sntb1	syntrophin, basic 1	-1.7360773	0.0226562
A0A075B5W2	A0A075B5W2	NA	-1.7608901	0.0203786
Q61285	Abcd2	ATP-binding cassette, sub-family D (ALD), member 2	-1.839118	0.0195527
O54774	Ap3d1	adaptor-related protein complex 3, delta 1 subunit	-1.8997958	0.0396109
A2AQ19	Rtf1	RTF1, Paf1/RNA polymerase II complex component	-1.9711807	0.0352119
P49290	Epx	eosinophil peroxidase	-1.9920238	0.0296583
P47941	Crkl	v-crk avian sarcoma virus CT10 oncogene homolog-like	-1.9991506	0.0304495
P10493	Nid1	nidogen 1	-2.0428692	0.0427194
Q99P88	Nup155	nucleoporin 155	-2.2311486	0.0339595
P58871	Tnks1bp1	tankyrase 1 binding protein 1	-2.2940625	0.016422
Q9Z1M8	Ik	IK cytokine	-2.3099211	0.0136874
P05064	Aldoa	aldolase A, fructose-bisphosphate	-2.32745	0.0386861
Q04857	Col6a1	collagen, type VI, alpha 1	-2.4355212	0.0288081
O88322	Nid2	nidogen 2	-2.4890993	0.0459355
Q8CGB6	Tns2	tensin 2	-2.8493697	0.0203617
C0HKD9	Mfap1a	microfibrillar-associated protein 1A	-2.9606212	0.0155524
Q61554	Fbn1	fibrillin 1	-3.5268239	0.0390791
P25444	Rps2	ribosomal protein S2	-3.6854786	0.0378189
Q91VM5	Rbmxl1	RNA binding motif protein, X-linked like-1	-4.0708897	0.0450577
Q80XU3	Nucks1	nuclear casein kinase and cyclin-dependent kinase substrate 1	-4.4330545	0.0124721

Comparison of fat samples with CK2α immunoprecipitations between distilled water- and MOTS-c administered mice (iPvalue < 0.05).

Supplemental-Table 12. Summary of human studies

Cohort	Outcome	Related Figure/table	Final sample number (n)	Female (%)	Number of m.1382A>C carriers (n, A/C allele)
Skeletal muscle gene expression	RNA sequencing	Figure 6A-D, S-Table 13-15	48	52.1	43/5
WASEDA'S Health Study	Sarcopenia risk* and ASMI by physical activity levels	Figure 6E-G, S-Tables 16-18	1241	33.4	1144/97
J-MICC (1st, baseline)	Prevalence of type 2 diabetes by age and physical activity levels	Figure 6H-J, S-Tables 19-20, and 22	11,853	58.1	10,959/894
J-MICC (2nd, 5 years later)	Skeletal muscle mass index and grip strength by age	S-Figure 13, S-Table 21	8226†	59.9	7596/630

ASMI: appendicular skeletal muscle index

*: Sarcopenia risk is assessed by ASMI <7.0 kg/m2 for men and <5.4 kg/m2 for women.

†: 8132 subjects had all the data for skeletal muscle mass index analysis.

Supplemental-Table 13. Characteristics of participants according to MOTS-c m.1382A>C in skeletal muscle gene expression cohort

	All				Men				Women			
MOTS-c m.1382A>C	A (n = 43)		C (N = 5)		A (n = 19)		C (N = 4)		A (n = 24)		C (N = 1)	
Variables	Mean	SD	Mean	SD	Mean	SD	Mean	SD	Mean	SD	Mean	SD
Women (%)	55.8		20.0									
Age (year)	25.7	7.5	24.0	4.7	26.6	8.2	24.8	5.1	24.9	7.0	21.0	-

Mean ± SD.

Supplemental-Table 15. Significant enriched KEGG pathways in Japanese carriers of the m.1382A>C polymorphism: Comparison of A (WT) or C (K14Q) allele carriers

Term ID	Description	LogP
hsa04510	Focal adhesion	-12.0542851
hsa04512	ECM-receptor interaction	-7.338889399
hsa04151	PI3K-Akt signaling pathway	-4.352705587
hsa05165	Human papillomavirus infection	-3.890558594
hsa05222	Small cell lung cancer	-2.711754969
hsa05146	Amoebiasis	-2.548784624
hsa05145	Toxoplasmosis	-2.416957717
hsa05133	Pertussis	-4.117399707
hsa04610	Complement and coagulation cascades	-2.819450524
hsa05171	Coronavirus disease - COVID-19	-2.651222387
hsa05322	Systemic lupus erythematosus	-2.096301118
hsa04974	Protein digestion and absorption	-3.495588834
hsa04360	Axon guidance	-4.052544011
hsa05100	Bacterial invasion of epithelial cells	-2.007459924
hsa05131	Shigellosis	-4.032724478
hsa04210	Apoptosis	-2.947123529
hsa05135	Yersinia infection	-2.93295888
hsa04750	Inflammatory mediator regulation of TRP channels	-2.61170303
hsa05415	Diabetic cardiomyopathy	-2.198824706
hsa04664	Fc epsilon RI signaling pathway	-2.155513301
hsa05417	Lipid and atherosclerosis	-2.096457221

Supplemental-Table 16. Characteristics of participants according to MOTS-c m.1382A>C in WASEDA'S Health Study

	All				Men				Women				P-value		
MOTS-c m.1382A>C	A (n = 1144)		C (N = 97)		A (n = 763)		C (N = 64)		A (n = 381)		C (N = 33)		MOTS-c m.1382A>C	Sex	Interaction
Variables	Mean	SD	Mean	SD	Mean	SD	Mean	SD	Mean	SD	Mean	SD			
Women (%)	33.3		34.0										0.644	NA	NA
Age (year)	55.2	9.9	56.6	10.2	56.9	10.3	59.1	10.8	52.0	8.4	51.7	6.9	0.375	**<0.001**	0.222
Height (cm)	166.5	7.8	166.4	8.2	170.3	5.8	170.6	6.0	158.9	5.3	158.3	5.2	0.948	**<0.001**	0.235
Body weight (kg)	63.9	11.4	63.5	11.7	68.8	9.5	68.6	10.1	54.0	7.6	53.7	7.9	0.894	**<0.001**	0.873
BMI (kg/m^2)	22.9	3.1	22.8	3.0	23.7	2.9	23.5	2.9	21.4	2.9	21.5	2.9	0.853	**<0.001**	0.678
ASMI (kg/m^2)	7.32	1.16	7.23	1.17	7.94	0.81	7.85	0.83	6.09	0.68	6.04	0.71	0.471	**<0.001**	0.963
ASM (kg)	20.5	4.5	20.2	4.6	23.1	2.9	22.9	3.0	15.4	2.0	15.1	2.1	0.594	**<0.001**	0.643
MVPA (min/week)	371	380	412	407	384	360	476	465	346	418	287	215	0.809	0.087	0.114
Low muscle mass (%)*	11.4		18.6		10.1		17.2		13.9		21.2		**0.043**	**0.032**	0.889

ASM, appendicular skeletal muscle mass; ASMI, appendicular skeletal muscle mass index; BMI, body mass index; MVPA, moderate to vigorous intensity physical activity.
Data were analyzed by two-way ANCOVA (MOTS-c m.1382A>C × sex) adjusted for age for continuous variables or logistic regression analysis (MOTS-c m.1382A>C × sex) adjusted for age for categorical variables.
*ASMI <7.0 kg/m^2 for men and <5.4 kg/m^2 for women.
Mean ± SD.

Supplemental-Table 17. Odds ratios for the prevalence of low muscle mass according to MOT-c m.1382A>C and MVPA group

Variables	OR (95% CI)	P-value
All subjects		
MOTS-c m.1382A>C (C allele)	1.75 (1.01, 3.02)	**0.044**
MVPA group (high MVPA group)	0.72 (0.49, 1.06)	0.094
Male subjects		
MOTS-c m.1382A>C (C allele)	1.76 (0.88, 3.52)	0.112
MVPA group (high MVPA group)	0.92 (0.53, 1.59)	0.764
Female subjects		
MOTS-c m.1382A>C (C allele)	1.72 (0.70, 4.19)	0.236
MVPA group (high MVPA group)	0.55 (0.31, 0.97)	**0.039**

Model was adjusted for age and sex

Supplemental-Table 18. Characteristics of participants according to MOTS-c m.1382A>C and MVPA group in WASEDA'S Health Study

MVPA group	Low (<150 min/week)				High (≥150 min/week)				P-value		
MOTS-c m.1382A>C	A (n = 281)		C (N = 25)		A (n = 863)		C (N = 72)		MOTS-c m.1382A>C	MVPA group	Interaction
	Mean	SD	Mean	SD	Mean	SD	Mean	SD			
All subjects											
Women (%)	42.7		36		30.2		33.3		0.664	0.017	0.391
Age (year)	52.4	9.1	54.0	10.9	56.2	10.0	57.5	9.9	0.236	0.004	0.939
Height (cm)	165.5	8.2	165.3	8.8	166.9	7.6	166.7	7.9	0.907	0.067	0.377
Body weight (kg)	63.8	12.7	61.5	11.6	63.9	10.9	64.2	11.8	0.309	0.504	0.058
BMI (kg/m^2)	23.2	3.7	22.3	2.7	22.9	2.9	23.0	3.2	0.239	0.887	0.081
ASM (kg)	19.9[†]	4.8	19.2	4.9	20.7	4.4	20.6	4.4	0.173	0.013	0.035
ASMI (kg/m^2)	7.19[†]	1.28	6.94	1.25	7.37	1.12	7.33	1.13	0.089	0.045	0.034
MVPA (min/week)	59	49	51	45	473	386	537	402	0.589	<0.001	0.370
Low muscle mass (%)*	13.9		24.0		10.5		16.7		0.044	0.094	0.764
Male subjects											
Age (year)	53.7	9.7	57.1	11.7	57.7	10.3	59.8	10.5	0.073	0.031	0.665
Height (cm)	170.5	6.2	169.8	6.8	170.3	5.7	170.8	5.7	0.584	0.211	0.561
Body weight (kg)	70.3	10.9	67.5	9.7	68.5	9.1	68.9	10.3	0.595	0.828	0.278
BMI (kg/m^2)	24.2	3.5	23.3	2.5	23.6	2.7	23.6	3.0	0.337	0.715	0.329
ASM (kg)	23.3	3.2	22.3	3.2	23.0	2.9	23.1	3.0	0.609	0.200	0.283
ASMI (kg/m^2)	8.00	0.92	7.71	0.78	7.92	0.78	7.89	0.85	0.317	0.405	0.316
MVPA (min/week)	66	49	52	45	469	359	617	456	0.297	<0.001	0.081
Low muscle mass (%)*	9.9		18.8		10.1		16.7		0.112	0.764	0.870
Female subjects											
Age (year)	50.6	8.0	48.3	6.6	52.7	8.4	52.9	6.7	0.546	0.045	0.462
Height (cm)	158.7	5	157.4	5.9	159	5.4	158.6	4.9	0.283	0.160	0.502
Body weight (kg)	55.1	9.2	50.9	4.9	53.5	6.8	54.8	8.6	0.336	0.375	0.068
BMI (kg/m^2)	21.9	3.5	20.6	1.9	21.2	2.5	21.8	3.1	0.605	0.818	0.114
ASM (kg)	15.4[†]	2.2	13.8	1.4	15.4	1.9	15.6	2.1	0.074	0.005	0.017
ASMI (kg/m^2)	6.09[†]	0.77	5.58	0.56	6.09	0.63	6.21	0.69	0.143	0.018	0.019
MVPA (min/week)	51	48	48	49	482	442	377	180	0.440	<0.001	0.503
Low muscle mass (%)*	19.2		33.3		11.5		16.7		0.236	0.039	0.772

ASM, appendicular skeletal muscle mass; ASMI, appendicular skeletal muscle mass index; BMI, body mass index; MVPA, moderate to vigorous intensity physical activity.
Data were analyzed by two-way ANCOVA (MOTS-c m.1382A>C × MVPA group) adjusted for age and sex for continuous variables or logistic regression analysis adjusted for age and sex for categorical variables.
*ASMI <7.0 kg/m^2 for men and <5.4 kg/m^2 for women.
†vs. C allele in the same MVPA group.
Mean ± SD.

Supplemental-Table 19. Characteristics of subjects in Saga area of J-MICC Study (1st study) by age groups.

Age groups	40s					50s					60s				
m.1382A/C	A		C		*P*	A		C		*P*	A		C		*P*
	Mean	SD	Mean	SD		Mean	SD	Mean	SD		Mean	SD	Mean	SD	
Male subjects															
(n)	1029		95			1,676		128			1,898		137		
Age (years old)	44.7	2.7	44.4	2.8	0.295	54.9	2.8	54.6	2.9	0.234	64.5	±2.8	64.8	2.9	0.352
Height (cm)	170.3	5.7	170.4	5.3	0.859	167.5	5.5	167.9	4.9	0.393	164.7	±5.7	164.6	5.3	0.870
BMI (kg/m^2)	24.3	3.2	24.7	3.5	0.246	24.3	3.0	24.4	3.2	0.697	24.1	±2.8	23.8	3.2	0.208
Waist circumference (cm)	85.3	8.2	85.7	9.3	0.675	86.4	7.8	86.4	8.6	0.981	86.6	±7.8	85.7	8.4	0.227
Smokers (%)	46.2%		48.4%		0.376	39.1%		39.8%		0.473	26.9%		30.7%		0.197
MVPA (min/day)	22.5	0.5	23.3	1.9	0.692	21.8	0.4	20.6	1.2	0.355	21.0	±0.4	22.8	2.4	0.452
Prevalence of type 2 diabetes (%)	5.3%		5.3%		0.603	10.4%		7.8%		0.224	14.2%		23.4%		0.004
Female subjects															
(n)	1695		136			2,335		192			2,326		206		
Age (years old)	44.6	2.8	44.7	2.9	0.815	54.9	2.8	54.6	3.0	0.227	64.3	2.7	64.4	2.9	0.685
Height (cm)	157.3	5.1	157.8	5.6	0.289	155.0	4.9	154.8	5.1	0.673	152.1	4.9	151.9	5.0	0.640
BMI (kg/m^2)	22.1	3.2	22.3	3.2	0.583	22.7	3.1	22.7	3.4	0.860	23.2	3.2	23.5	3.4	0.328
Waist circumference (cm)	77.7	8.8	79.0	8.7	0.082	81.0	9.1	80.6	9.9	0.482	83.9	9.3	83.7	10.0	0.757
Smokers (%)	10.9%		14.7%		0.111	10.4%		11.5%		0.353	4.4%		3.9%		0.460
MVPA (min/day)	19.4	0.3	18.5	1.0	0.358	19.6	0.3	18.8	0.9	0.393	17.5	0.3	16.1	1.0	0.145
Prevalence of type 2 diabetes (%)	1.4%		0.7%		0.458	4.5%		1.6%		0.029	7.3%		7.8%		0.446

BMI, body mass index. MVPA, moderate to vigorous physical activity. Mean ± SD.

Supplemental-Table 20. Statistical Model Analysis of Type 2 Diabetes Risk Based on Interaction of Multiple Factors among Subjects in the Saga Area of the J-MICC Study (1st Study).

Model	Variables	Coefficient	Standard error	Confidence interval	P	Adjusted R^2	AIC
Model 1						0	1706.0
	Intercept	0.073	0.0024	0.068, 0.078	**<0.001**		
	MOTS-c m.1382A>C	0.003	0.0090	-0.016, 0.020	0.806		
Model 2						0.030	1349.9
	Intercept	-0.160	0.0250	-0.209, -0.110	**<0.001**		
	Age	0.005	0.0004	0.004, 0.006	**<0.001**		
	Sex	0.030	0.0330	-0.034, 0.095	0.356		
	Age * Sex	-0.002	0.0006	-0.003, -0.004	**0.005**		
Model 3						0.030	1350.7
	Intercept	-0.149	0.0425	-0.232, -0.066	**<0.001**		
	Age	0.005	0.0007	0.003, 0.006	**<0.001**		
	Sex	0.046	0.0567	-0.066, 0.157	0.422		
	MVPA	0.000	0.0015	-0.003, 0.003	**0.761**		
	Age * Sex	-0.002	0.0010	-0.0040, -0.0002	**0.033**		
	Age * MVPA	0.00001	0.000026	-0.00004, 0.00006	0.804		
	Sex * MVPA	-0.001	0.0023	-0.006, 0.003	0.624		
	Age * Sex * MVPA	0.000030	0.000040	-0.00005, 0.00011	0.437		
Model 4						0.030	1345.4
	Intercept	-0.142	0.0265	-0.194, -0.090	**<0.001**		
	Age	0.004	0.0005	-0.416, -0.053	**<0.001**		
	Sex	0.015	0.0344	0.004, 0.005	0.654		
	MOTS-c m.1382A>C	-0.235	0.0927	-0.052, 0.083	**0.011**		
	Age * Sex	-0.001	0.0006	0.001, 0.008	**0.032**		
	MOTS-c m.1382A>C * Age	0.005	0.0016	-0.043, 0.434	**0.004**		
	MOTS-c m.1382A>C * Sex	0.195	0.1215	-0.002, 0.0001	0.108		
	MOTS-c m.1382A>C * Age * Sex	-0.004	0.0022	-0.008, 0.00007	0.054		
Model 5						0.032	1336.7

Intercept	-0.096	0.045	-0.184, -0.008	**0.032**
Age	0.004	0.001	0.002, 0.005	**<0.001**
Sex	-0.004	0.060	-0.121, 0.112	0.941
MVPA	-0.002	0.002	-0.005, 0.001	0.204
MOTS-c m.1382A>C	-0.498	0.142	-0.776, -0.220	**<0.001**
Age * Sex	-0.001	0.001	-0.003, 0.001	0.283
Age * MVPA	0.00004	0.00003	-0.00002, 0.00009	0.177
Sex * MVPA	0.001	0.002	-0.004, 0.005	0.831
MOTS-c m.1382A>C * Age	0.010	0.002	0.005, 0.015	**<0.001**
MOTS-c m.1382A>C * Sex	0.468	0.197	0.081, 0.854	**0.018**
MOTS-c m.1382A>C * MVPA	0.012	0.005	0.002, 0.021	**0.015**
Age * Sex * MVPA	-0.000001	0.00004	-0.0001, 0.0001	0.983
MOTS-c m.1382A>C * Age * Sex	-0.009	0.003	-0.016, -0.003	**0.005**
MOTS-c m.1382A>C * Age * MVPA	-0.0002	0.0001	-0.0004, -0.0001	**0.004**
MOTS-c m.1382A>C * Sex * MVPA	-0.013	0.008	-0.028, 0.003	0.124
MOTS-c m.1382A>C * Age * Sex * MVPA	0.0002	0.0001	-0.00003, 0.0005	0.081

AIC, akaike information criterion. MVPA, moderate to vigorous physical activity (min/day).

Supplemental-Table 21. Skeletal muscle mass index and grip strength of subjects in Saga area of J-MICC Study (2nd study) by m.1382A>C polymorphism divided with age groups.

Age groups	40-49 years at baseline (45-54 years at the 2nd survey)					50-59 years at baseline (55-64 years at the 2nd survey)					60-69 years at baseline (65-74 years at the 2nd survey)				
m.1382 A/C	A		C		*P*	A		C		*P*	A		C		*P*
	Mean	SE	Mean	SE		Mean	SE	Mean	SE		Mean	SE	Mean	SE	
Male subjects															
	632		54			1162		92			1344		98		
Skeletal muscle mass index (kg/m^2)	7.68	0.01	7.73	0.03	0.179	7.14	0.01	7.16	0.02	0.422	6.55	0.10	6.49	0.02	0.011
Grip strength (kg)	41.3	0.2	42.6	0.8	0.110	38.6	0.2	38.9	0.6	0.615	34.9	0.1	33.9	0.5	0.076
Female subjects															
N	1165		95			1646		144			1647		147		
Skeletal muscle mass index (kg/m^2)	7.16	0.01	7.15	0.02	0.847	6.67	0.01	6.71	0.02	0.061	6.21	0.01	6.21	0.02	0.968
Grip strength (kg)	25.5	0.1	26.0	0.4	0.266	23.5	0.1	23.7	0.3	0.669	22.0	0.1	22.3	0.3	0.303

Mean ± SE. Values are adjusted by body mass index (BMI) and moderate-to-vigorous intensity physical activity (MVPA).

Supplemental-Table 22. Characteristics of subjects in Saga area of J-MICC Study (1st study) by physical activity levels in 60s

MVPA (min/week)	Low (<150 min/week)					High (≥150 min/week)				
MOTS-c 1382C>A	A		C		*P*	A		C		*P*
Male subjects	Mean	SE	Mean	SE		Mean	SE	Mean	SE	
n	1211		89			687		48		
Age (years)	64.6	0.1	64.7	0.3	0.614	64.5	0.1	64.8	0.4	0.379
Height (cm)	164.9	0.2	164.3	0.6	0.319	164.2	0.2	165.1	0.8	0.275
BMI (kg/m^2)	24.1	0.1	23.9	0.4	0.447	24.0	0.1	23.6	0.4	0.254
Body fat (%)	23.1	0.1	23.1	0.5	0.872	22.1	0.2	22.0	0.6	0.847
MVPA (min/week)	70.0	1.2	60.8	4.4	0.037	282.4	5.1	343.2	33.8	0.082
Smokers (%)	31.3		33.7		0.637	19.2		25		0.347
Type 2 diabetes (%)	13.5		31.5		<0.001	15.4		8.3		0.214
Female subjects										
n	1662		155			664		51		
Age (years)	64.4	0.1	64.4	0.2	0.913	63.9	0.1	64.1	0.4	0.653
Height (cm)	152.4	0.1	152.1	0.4	0.476	151.5	0.2	151.6	0.6	0.899
BMI (kg/m^2)	23.2	0.1	23.5	0.3	0.393	23.2	0.1	23.4	0.4	0.647
Body fat (%)	32.7	0.1	32.9	0.5	0.645	32.4	0.2	32.3	0.8	0.819
MVPA (min/week)	72.6	0.9	67.3	3.0	0.095	248.5	3.8	250.1	12.5	0.908
Smokers (%)	4.8		5.2		0.844	3.3		0		0.392
Type 2 diabetes (%)	6.5		8.4		0.397	9.3		5.9		0.612

Mean ± SE. BMI, body mass index. MVPA, moderate to vigorous physical activity.
